# Structured Cardiac Assessment and Treatment Following Exacerbations of COPD (SCATECOPD): A Pilot Randomised Controlled Trial

**DOI:** 10.3390/biomedicines13030658

**Published:** 2025-03-07

**Authors:** Joseph Kibbler, Eduwin Pakpahan, Stephen McCarthy, Rebecca Webb-Mitchell, Arun Prasad, David P. Ripley, Joanne Gray, Stephen C. Bourke, John Steer

**Affiliations:** 1 Department of Respiratory Medicine, Northumbria Healthcare NHS Foundation Trust, North Shields NE29 8NH, UK; 2 Translational and Clinical Research Institute, Newcastle University, Newcastle upon Tyne NE1 7RU, UK; 3 Applied Statistics Research Group, Department of Mathematics, Physics & Electrical Engineering, Northumbria University, Newcastle upon Tyne NE1 8ST, UK; 4 Faculty of Health & Life Science, Northumbria University, Newcastle upon Tyne NE1 8ST, UK; 5 School of Medicine, Faculty of Health Sciences and Wellbeing, University of Sunderland, Sunderland SR1 3SD, UK

**Keywords:** COPD, comorbidity, multimorbidity, heart failure, coronary artery disease

## Abstract

**Background/Objectives**: Heart disease is common in COPD, yet it is underdiagnosed and undertreated. Heart failure (HF) is undiagnosed in up to 20% of hospital inpatients. Hospitalised exacerbations of COPD (ECOPD) confer high mortality and readmission rates, with an elevated temporal cardiac risk. We performed a pilot randomised controlled trial examining the feasibility and effect of inpatient structured cardiac assessment (SCA) to diagnose and prompt guideline-recommended treatment of heart disease. **Methods**: A total of 115 inpatients with ECOPD were randomised 1:1 to receive usual care (UC) or SCA, comprising transthoracic echocardiography, CT coronary artery calcium scoring, 24 h ECG, blood pressure, and diabetes assessment. Follow-up was for 12 months. The prevalence of underdiagnosis and undertreatment of heart disease were captured, and potential outcome measures for future trials assessed. **Results**: Among patients undergoing SCA, 42/57 (73.7%) received a new cardiac diagnosis and 32/57 (56.1%) received new cardiac treatment, compared with 11/58 (19.0%; *p* < 0.001) and 5/58 (8.6%; *p* < 0.001) in the UC group. More patients in the SCA group were newly diagnosed with HF (36.8% vs. 12.1%; *p* = 0.002). When heart disease was diagnosed, the proportion receiving optimal treatment at discharge was substantially higher in SCA (35/47 (74%) vs. 4/11 (34%); *p* = 0.029). The occurrence of a major adverse cardiovascular event (MACE) showed promise as an appropriate clinical outcome for a future definitive trial. MACEs occurred in 17.2% in usual care vs. 10.5% in SCA in one year, with a continued separation of survival curves during follow up, although statistical significance was not shown. **Conclusions**: A structured cardiac assessment during ECOPD substantially improved diagnosis and treatment of heart disease. HF and coronary artery disease were the most common new diagnoses. Future interventional trials in this population should consider MACEs as the primary outcome.

## 1. Introduction

Chronic obstructive pulmonary disease (COPD) robs the global population of millions of years of healthy life due to excess mortality and disability [1]. COPD exacerbations (ECOPD) are a major cause of mortality and hospital admissions [2]. In the UK, inpatient mortality has fallen from 7.4% in 2003 to 3.5% in 2019 across national audits [3,4]. However, the reduction in 90-day mortality over the same period has been more modest, from 15.3% to 12%. Simultaneously, 90-day readmission rates have risen, from 31.4% to 43% [5].

COPD typically occurs in the context of multimorbidity [6], with disease clusters including cardiometabolic, neuro-psychiatric, and musculoskeletal conditions identified [7]. Co-existing conditions are highly impactful: a UK cohort study of 67,516 patients found that non-COPD causes accounted for 61.3% of deaths. Non-COPD conditions are also the cause of 60.5% of readmissions [5].

Cardiovascular disease (CVD) is strongly associated with COPD, occurring approximately twice as often in patients with COPD than those without (adjusted relative risk [RR] for unspecified CVD 1.6–2.7) [8]. Heart failure (HF) specifically is two to four times more probable in COPD patients (adjusted RR 1.8–3.9) and is diagnosed six times more frequently than in matched controls (incidence rate ratio [IRR] 5.94 (5.50–6.42) [9]. Comorbid heart disease adversely affects outcomes: a meta-analysis of nine studies including 6133 patients showed that patients with both COPD and heart disease were hospitalised more frequently (rate ratio 1.56 [1.52–1.60]) and were at approximately 50% higher risk of dying from any cause (hazard ratio [HR] 1.61 [1.38–1.83]) [10].

Major adverse cardiovascular events (MACEs) are particularly common in the immediate post-exacerbation period. The latest data from primary care and hospital registries show that heightened risk continues for up to 1 year post-exacerbation [11]. A UK self-controlled case series (n = 5696) revealed that, during the first 3 days following hospitalisation, myocardial infarction (MI) occurred 8 times more frequently than during the control period pre-exacerbation (IRR = 8.00, 5.81–11.01). In the same study, over this period, ischaemic stroke occurred 3 times as frequently (IRR = 3.39, 1.52–7.58) [12].

Major guidelines have advocated investigating patients when specific symptoms and signs of heart disease are present [13,14]. The latest GOLD report also suggests considering the use of diagnostic biomarkers (troponin and NT-proBNP, which are released from cardiac myocytes in response to ischaemia and stretch, respectively) to assess for the contribution of heart failure, MI, and arrhythmia to suspected ECOPD [15]. However, heart disease and COPD cause similar symptoms, and this approach is clearly insufficient. This was shown in a US cohort where, despite similarly high rates of MACEs to other cohort studies, only 2.2% of patients received a formal cardiac risk assessment [16]. As a consequence, the underdiagnosis of CVD is rife. A proactive investigation for cardiac disease in patients with COPD in a community setting found undiagnosed HF in 20.3% of patients. Over half of these patients had left ventricular systolic dysfunction (LVSD) with an LVEF (left ventricular ejection fraction) below 45% [17]. In a study of hospitalised patients with ECOPD, severe coronary artery disease (CAD) was diagnosed in 18% and severe LVSD in 8% [18]. Importantly, in the latter study, clinical examination and diagnostic biomarkers did not help to differentiate the presence of severe CAD and LVSD from normality. This further emphasises the diagnostic challenge present in this population.

Even when CVD is diagnosed, undertreatment is common. In a Danish COPD registry of patients with coexistent heart failure, only 52% of patients were prescribed beta-blockers, compared with 89% of HF patients without COPD [19]. Observational studies of patients with COPD have suggested that those in receipt of beta-blockers or statins had better outcomes [20]. However, prospective RCTs in which patients with known indications for beta-blockers and statins were excluded have shown that the use of these drugs when a relevant CVD comorbidity is absent is ineffective and potentially harmful [21,22]. This highlights that underdiagnosis and undertreatment are key causes of the high rates of adverse cardiovascular outcomes in COPD. Proactive and rigorous strategies to both diagnose and optimise the treatment of CVD are needed. The marked increase in MACE risk following hospitalisation for ECOPD suggests this is the opportune moment for intervention.

We examined, in a randomised controlled trial of patients hospitalised for ECOPD, the impact of a predefined structured cardiac assessment (with prompt guideline-directed treatment of identified heart disease) compared to usual care. To our knowledge, this is the first prospective trial involving both the diagnosis and treatment of heart disease in this population. Key outcome measures were the rates of diagnosed heart disease, the proportion of optimally treated heart disease, and patient outcome post-discharge. The trial objectives were as follows:

(1) To quantify the disease burden in the population studied and compare the diagnostic yield of structured cardiac assessment (SCA) and usual care (UC);
(2) To quantify the scale of undertreatment of heart disease;
(3) To assess the effect of SCA versus UC on patient outcomes in order to identify optimal outcome measures for future interventional trials.

## 2. Materials and Methods

### 2.1. Study Design and Participants

The Structured Cardiac Assessment and Treatment following Exacerbations of COPD (SCATECOPD) study was a pilot randomised controlled trial performed at a single UK centre. Those assigned to structured cardiac assessment (SCA) received, as soon as practicable following inpatient randomisation, a predetermined set of tests to diagnose heart disease. Conditions identified by SCA were managed by independent treating clinicians according to the contemporary national or local guidelines. Management summaries were created for common conditions to help clinicians and ensure consistent treatment approaches. The control group (usual care) received standard inpatient and follow-up care for ECOPD. Any cardiac tests performed in the usual care group were according to the individual treating clinicians’ decisions and not part of the study protocol. The full trial protocol and management summaries are found in online supplemental Appendix A and Appendix B.

Patients were recruited to this study on a non-consecutive but systematic basis. Patients admitted with ECOPD were assessed for eligibility based on order of admission until a weekly recruitment target of up to 3 patients was met. Follow-up was face-to-face at 90 days and 12 months and by telephone at 6 and 9 months. Inclusion criteria were designed to be broad to ensure the study population would be generalisable and were as follows:

(1) Age > 35 years;
(2) More than 10 pack-year history of tobacco smoking;
(3) Clinical diagnosis of COPD;
(4) Previous obstructive spirometry (FEV1/FVC < 0.7);
(5) Hospitalisation with primary cause being ECOPD.

Exclusion criteria were as follows:

(1) Inability to provide informed consent;
(2) Any non-COPD condition likely to limit survival to <12 months;
(3) Contra-indication to cardiac CT, including inability to lie flat;
(4) Pregnancy or breastfeeding.

Written informed consent was required, with ethical approval granted by the East of Scotland Research Ethics Service (local reference 20/ES/0112). Trial registration was at ISRCTN (ISRCTN26935612).

### 2.2. Randomisation

Eligible patients were randomised to SCA or usual care (UC) in a 1:1 ratio using stratified, block randomization, with the aid of an online platform (sealedenvelope.com). Block sizes of 4 and 2 were used to maximise the probability of equal sample sizes in a small study with multiple strata. The stratification factors were (1) presence or absence of known significant heart disease (any of the following: HF with LVEF < 45%; MI or CAD requiring revascularisation; atrial fibrillation) and (2) likelihood of 90-day readmission or death as predicted by the PEARL score [23], classified as low (0–1), medium (2–4) or high (5–9). PEARL is a predictor of 90-day readmission or death and comprises five indices: Previous admissions, Extended MRC dyspnoea score (eMRCD) score [24], Age, Right-sided heart failure, and Left-sided heart failure [23]. Allocation was not blinded, although where independent assessments were undertaken (for example, adjudication of cause of death), assessors were blinded to allocation.

### 2.3. Structured Cardiac Assessment

The investigations comprising SCA were (1) bedside transthoracic echocardiography performed by an accredited sonographer, with intravenous contrast (Sonovue^TM^, Bracco S.p.A., Milan, Italy) used if necessary for quantitative LVEF measurement; (2) non-contrast cardiac CT for calculation of coronary artery calcium score (CACS, which correlates with advanced atherosclerosis and hence cardiovascular event risk); (3) 24 h 3-lead ECG; (4) blood pressure assessment; (5) blood tests, including lipid profile and HbA_1C_.

Due to the lack of a single, universally employed classification system at the time of this study, the following approach was taken towards HF diagnosis: patients were diagnosed with HF with moderate–severe LVSD if LVEF < 45% and HF without moderate–severe LVSD if LVEF ≥ 45%, with echocardiographic evidence of LV diastolic dysfunction. Right-sided HF was diagnosed if right ventricular function was impaired alongside clinically evident peripheral oedema; this could co-exist with either of the above diagnoses of LV dysfunction.

We synthesised relevant guidelines and local practices into management summaries in summer 2020 (prior to the publication of evidence supporting the use of sodium-glucose co-transporter 2 (SGLT2) inhibitors in HF with LVEF above 40% [25,26]). Noteworthy advice included the initiation of a beta blocker and ACE inhibitor and referral to a local HF service for patients with moderate–severe LVSD and aspirin 75 mg daily for patients with CACS > 100 (see Supplementary Section S2 for details).

### 2.4. Outcomes

Multiple outcomes were examined to evaluate for feasibility and for differences, both numerical and statistical, that would suggest suitability for assessment by a definitive RCT. As this was a pilot study, conclusions about effects on a primary outcome were not sought. Nevertheless, we prespecified days alive outside hospital (DAOH) over 12 months post-discharge as our main outcome of interest, because it combines the important outcome measures of mortality, readmission, and length of stay.

Additional outcomes included proportions of patients who received diagnoses of CVD that were not known prior to study entry (new CVD); prevalence of undertreated CVD (i.e., the proportion of patients who lacked guideline-recommended treatment for diagnosed conditions; see Supplementary Section S2 for pre-specified summaries of management of CVD); time to readmission (or death without readmission); all-cause readmission rates at 90 days and 12 months; all-cause mortality rates at 90 days and 12 months; COPD exacerbation rates, from health records and self-report, at 90 days and 12 months; rates of traditional three-point MACEs (nonfatal stroke, MI, or cardiovascular death) at 90 days and 12 months; and mean change in quality of life measured by St. George’s Respiratory Questionnaire for COPD (SGRQ-C) over 12 months.

### 2.5. Statistical Analysis

#### 2.5.1. Sample Size

This pilot study was performed to assess the feasibility of a definitive multicentre trial, including recruitment, selection of the primary outcome, and data to support a power calculation. A pragmatic recruitment target of 120 patients was set. This was based on ECOPD admission rates and recruitment rates for studies of a similar nature that had previously been conducted at the study centre.

#### 2.5.2. Outcome Analysis

Analysis of outcomes was on an intention-to-treat basis. DAOH was calculated for all randomised patients. For main outcome analysis, the distribution of DAOH for each study arm was compared using Mann–Whitney U test.

For additional outcomes, tests for difference between the study arms were carried out using Student’s *t*-test for comparing means; the Mann–Whitney U test for data that were non-normal by the Shapiro–Wilk test; and Fisher’s exact test for categorical data. Tests for change between time points (baseline, 90 days, and 12 months) were carried out using paired Student’s *t*-test to compare means and the Wilcoxon signed-rank test for non-parametric data. Time to event outcomes was assessed by the Kaplan–Meier method and differences tested by log-rank test. Quality of life was analysed using the area-under-the-curve method, with time-weighted change in quality of life compared between the study arms using Student’s *t*-test. Sample size calculations for potential future studies were carried out based on observed effect sizes for outcomes of interest, using a two-tailed α = 0.05 and type II error rate of 0.2.

All statistical tests were two-tailed, with *p* < 0.05 considered statistically significant.

Calculations were carried out using SPSS Statistics version 28 (IBM, Armonk, NY, USA), Stata version 17 (StataCorp, College Station, TX, USA), and the UCSF Clinical and Translational Science Institute sample size calculator [27].

## 3. Results

**Figure 1:**
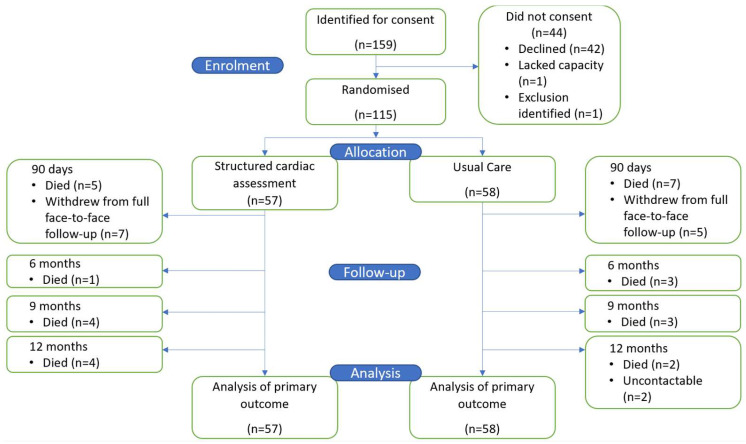
Study flow chart.

A total of 159 patients were identified as suitable for enrolment by their clinical team between 17 December 2020 and 30 May 2022. Recruitment was slower than anticipated due to the COVID-19 pandemic. A total of 115 patients were both eligible and provided consent; 57 patients were allocated to the SCA arm and 58 to UC (Figure 1). Trial recruitment was ceased in May 2022 after recommendation from the Trial Management Group that sufficient numbers had been recruited to meet trial objectives.

### 3.1. Baseline Demographics

Patient demographics were similar to UK National Audit data [25]. Mean age was 72.0 (SD 6.4) years and mean FEV1 49.1 (SD 18.3) percent predicted. Most patients experienced frequent COPD exacerbations (median [IQR] 3 [1,2,3,4,5] in the past year). A total of 44% were housebound due to breathlessness (median [IQR] eMRCD 4 [4–5a]) and 87% had mild-to-moderate frailty (Rockwood clinical frailty score [CFS] ≥ 5, indicating a need for help with daily activities [28]). At study entry, hypertension was the commonest known diagnosis, occurring in approximately half of the patients. One in seven patients had previously had an MI and one in five a stroke or transient ischaemic attack.

Index exacerbations were generally of low–intermediate severity according to DECAF score [29] (median [IQR] DECAF = 2 [1,2]). Acute non-invasive ventilation was used in 21.7% of cases. Two patients died between allocation and discharge, both in UC. Statistical differences in baseline patient characteristics (Table 1) were non-significant, although the numbers involved were small. The largest observed discrepancies were in baseline rates of angina (17.5% SCA, 8.6% UC) and moderate–severe LVSD (1.7% SCA and 5.3% UC).

By 12 months, 29 patients (25.2%) had died: 14 in SCA and 15 in UC. Additionally, eight patients in SCA and seven patients in UC dropped out from full review (Figure 1). No patient withdrew consent for their hospital records to be accessed until the end of follow-up for the collection of admission and mortality data.

### 3.2. Heart Disease Diagnosis and Treatment

#### 3.2.1. Heart Failure

Patients in SCA had more tests to identify HF: 56/57 (98.2%) had NT-proBNP testing and 55/57 (96.5%) had echocardiography, compared with 14/58 (24.1%) and 16/58 (25.9%), respectively, in UC. Correspondingly, a greater proportion of patients in SCA were newly diagnosed with HF (36.8% vs. 12.1%; *p* = 0.002).

NT-proBNP levels were raised above 300 pg/mL (the ESC-recommended cutoff for excluding heart failure in acutely unwell patients) in 36/56 (64.3%). Thirty-four of these patients underwent echocardiography and moderate–severe LVSD was present in seven (20.6%), a false-positive rate for NT-proBNP > 300 pg/mL of 79.4%. One of twenty patients who had both normal NT-proBNP and echocardiography had moderate–severe LVSD, a false-negative rate of 5%. For all HF diagnoses, the false-positive rate was 50% (14/28 had raised NT-proBNP and no HF) and the false-negative rate was 23.1% (6/26 had normal NT-proBNP and HF).

**Figure 2:**
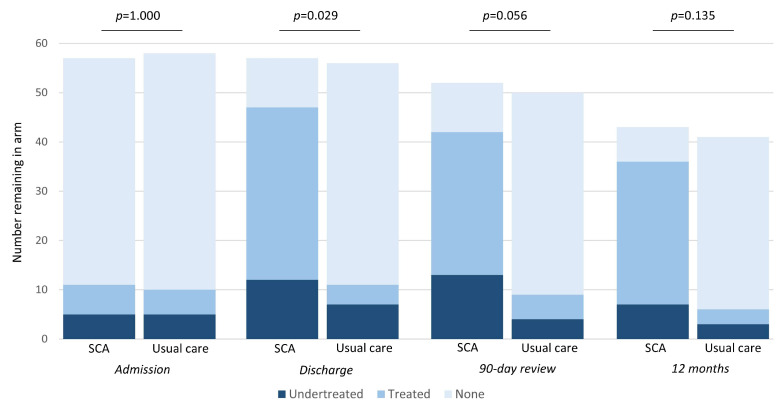
Numbers with treated and undertreated heart disease during study progress. *p* values are from Fisher’s exact test for difference in proportions of undertreated heart disease at each time point. This proportion is equal to the treated number (mid-blue bar) divided by the total number with heart disease (mid-blue bar plus dark blue bar).

During 12 months of follow-up, most patients in UC did not narrow their diagnostic deficit and undergo cardiac investigations at a later date (see Figure 2): 21/56 (37.5%) of patients in UC that survived to discharge underwent echocardiography, leading to delayed HF diagnoses in 6 cases.

#### 3.2.2. Other Cardiovascular Disease

Tests for other cardiovascular diseases were performed more frequently in SCA. In the SCA arm, 54/57 (94.7%) had troponin tested and 55/57 (96.5%) had a lipid profile, compared with 9/58 (15.5%) and 1/58 (1.7%) in UC.

**Figure A1:**
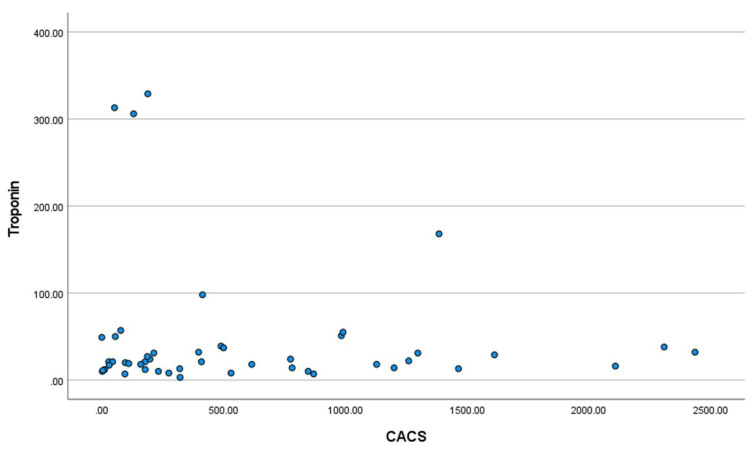
Scatter-plot of Troponin T at admission with ECOPD and coronary artery calcium score (CACS). Pearson correlation coefficient −0.112; *p* = 0.449.

Troponin levels were above the 99th percentile (14 ng/L) in 39/54 (72.2%). The proportion with moderate–severe CAD was similar for patients with raised (65.8%) and normal (73.3%) troponin levels. There was no correlation between CACS and troponin level (Pearson correlation coefficient −0.112; *p* = 0.449. See Figure A1, Appendix B).

Overall, a substantially higher proportion of patients in SCA had a new diagnosis of a CVD by the conclusion of SCA than those receiving usual care: 42/57 (73.7%) compared with 11/58 (19.0%; *p* < 0.001) in UC (see Table 2). Moderate–severe CAD was the most common diagnosis in SCA (59.6% vs. 0% in UC; *p* < 0.001; Table 2).

In the 12 months post-discharge, only a small number of diagnoses of CV disease, besides HF, were made in UC (one of CAD, one of hypertension; two patients suffered MI), indicating that effective investigation did not take place on a delayed basis in this group.

SCA identified that 48.3% of patients with an existing diagnosis of hypertension and 44.4% of those with diabetes had suboptimal control. Furthermore, while there were equal proportions of patients with known heart disease in each arm at admission, there was a significantly lower rate of undertreatment in SCA following hospital discharge: 25.5% vs. 63.6% in UC (*p* = 0.029; Figure 2). This implies that, even in those patients known to have heart disease, treatment was not being optimised as part of usual care during index hospital admission. The most common medications started following SCA were antiplatelets and beta-blockers (statins, where indicated, were generally already prescribed). The rate of undertreatment did not significantly narrow during follow-up and remained higher for UC (Figure 2). This indicates that throughout the study period, for those receiving usual care, cardiac diagnoses remained undertreated and were not addressed at outpatient follow-up visits.

### 3.3. Clinical Outcomes

Readmission and mortality rates were high during follow-up, in line with UK national audit data [5], at 44.3% and 25.2% at 12 months, respectively. Mortality rates were similar between arms at 90 days (SCA = 3 [5.3%] vs. UC = 5 [8.6%]) and 12 months (SCA = 14 [24.6%] vs. UC = 15 [25.9%]. However, within SCA, if patients had moderate–severe LVSD, 12-month mortality was doubled: 4/7 (57%) compared with 10/48 (21%) without the diagnosis. This was short of statistical significance by Fisher’s exact test, with *p* = 0.061.

Mortality adjudication determined that the most common cause of death was COPD (20/29 patients), followed by CVD (5 cases).

**Figure 3:**
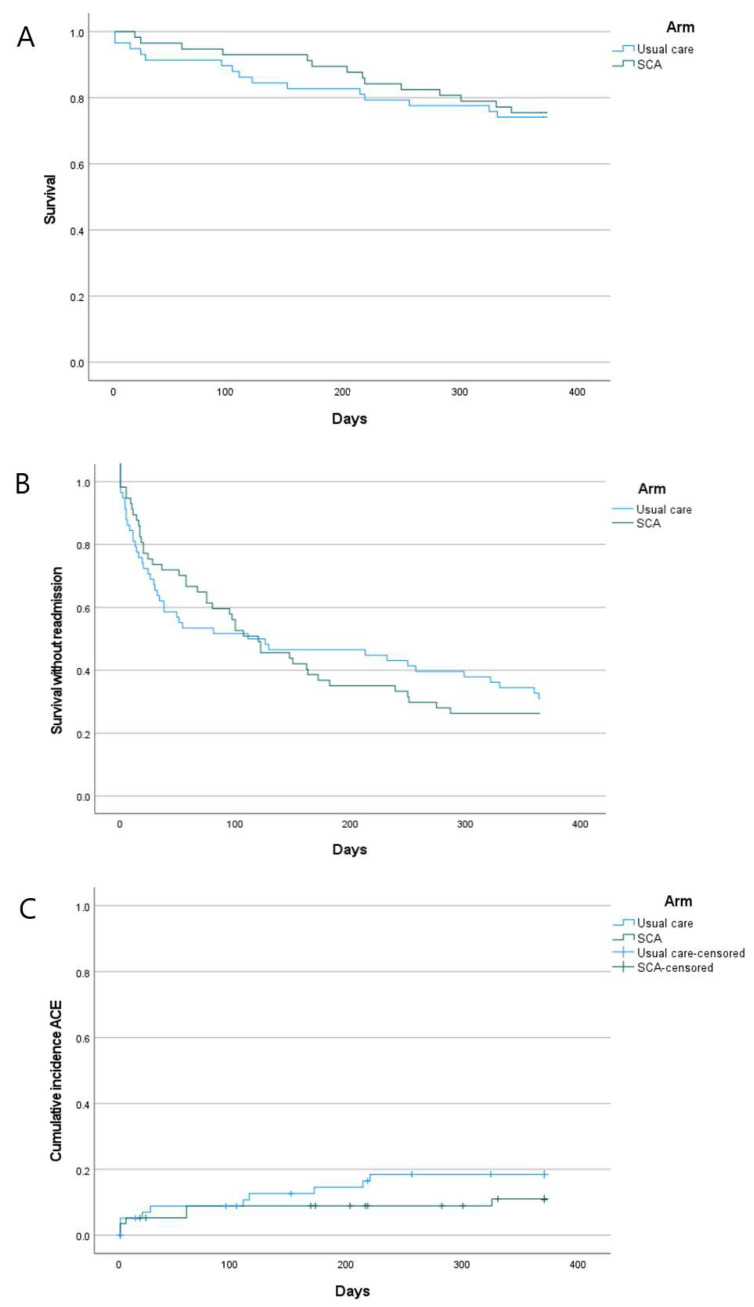
Kaplan–Meier plots. (**A**): Mortality; (**B**): survival without readmission; (**C**): incidence of major adverse cardiovascular events (ACEs). Censored patients in (**C**) are those who dropped out of follow-up for reasons other than experiencing an ACE.

There was no significant difference in time to death (HR = 0.903 [95% CI 0.436–1.871], *p* = 0.783; Figure 3A), although on Kaplan–Meier plots, which display changes in survival probability over time, the curves remained separated. The HR suggests a sample size of 7754 would be required to adequately power a study with 2-year follow-up using time to death as a primary outcome [27] (see Appendix A for calculation details). Readmission rates were comparable: 45.6% in SCA and 43.1% in UC at 90 days and 73.7% in SCA and 63.8% in UC at 12 months. An early difference in survival without readmission was seen, with lower rates in SCA, but with time, the survival curves crossed, with no difference in risk across the follow-up period (HR = 1.053 [0.682–1.627], *p* = 0.81) (Figure 3B).

**Figure A2:**
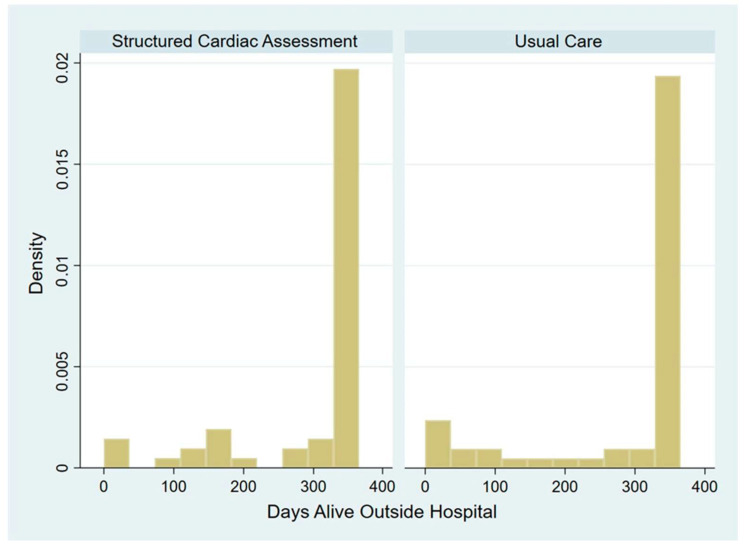
Histogram showing the distribution of DAOH by study arm.

Most patients experienced a small reduction in DAOH from the maximum value of 365; hence, the distribution of DAOH was highly negatively skewed (see Figure A2, Appendix B). There was no apparent difference between the arms, with a median DAOH of 356 (IQR 284–365) in SCA and 356 (IQR 313–365) in UC.

Quality of life, as measured by SGRQ-C, worsened overall during study follow-up, i.e., the mean SGRQ-C score increased. No significant difference existed between the study arms: in SCA, the time-weighted mean change in SGRQ-C was +1.02 (SD 9.90), and in usual care, it was +2.24 (SD 10.45; *p* = 0.533).

Major adverse cardiovascular events (MACEs) over 12 months were observed in 10 patients in UC (17.2%) and 6 in SCA (10.5%). Analysis of time to MACE (censoring patients who died for non-cardiovascular reasons) revealed a separation of the curves, with the cumulative incidence of MACEs higher in UC, albeit not significantly (HR = 0.582 [0.211–1.601], *p* = 0.294, Figure 3C). A 1:1 RCT with 80% power to detect this difference, with 2 years of follow-up, would need to recruit 528 participants [27] (see Appendix A for calculation details).

## 4. Discussion

In this pilot study, a structured cardiac assessment, when applied to all patients irrespective of clinical history or treating clinician’s assessment, identified a considerable burden of undiagnosed heart disease. A total of 74% of patients received a new diagnosis during hospital admission, compared with 19% in UC. HF (either left- or right-sided) was newly diagnosed in 36.8% of patients undergoing SCA. Patients with significant LVSD had twice the one-year mortality of those without. Additionally, a majority of patients (59.6%) had newly diagnosed high levels of coronary artery calcification (CACS > 100).

Importantly, SCA identified disease amenable to effective treatment: 56% received new evidence-based treatment during the index hospital admission, compared with just 9% in UC. As a result, the proportion of patients with heart disease that was not optimally treated was significantly reduced in SCA, from 45.5% at admission to 25.5% at discharge. Remaining undertreatment was predominantly due, in 73.3% of cases, to drug intolerance/contraindication or patient choice. The importance of prompt diagnosis and treatment was further highlighted by the low numbers of patients in UC who had heart disease diagnosed and treated following hospital discharge. The diagnostic deficit in patients receiving standard care was not ‘caught up’ and clinicians remained ignorant to the presence of undiagnosed heart disease.

Our results call into question COPD guideline statements advising that investigation for cardiac disease should be prompted by clinical assessment of symptoms and signs. Patients receiving usual care (cardiac tests organised at treating clinicians’ discretion) were much less likely to be found to have a new cardiac disease than those who underwent the structured inpatient cardiac assessment (19% vs. 73.7%). Furthermore, differences in rates of underdiagnosis and undertreatment between SCA and UC did not substantially narrow during 12-month follow-up. ECOPD is therefore a critical moment to intervene to improve cardiovascular disease care, but clinicians do not have effective guidance on how to do so. For example, if the tentative suggestions in GOLD 2025 to use diagnostic biomarkers were followed universally [15], our data suggest that the majority of inpatients should be investigated in more detail, because troponin was elevated in 72.2% and NT-proBNP in 64.3%. This approach would still fail to identify 23% of HF and 26.7% of moderate–severe CAD, however. We believe that the current approach of investigating for the presence of cardiovascular disease on the basis of symptoms, signs, or inaccurate biomarkers needs to be discontinued. Instead, a structured approach based on risk of cardiovascular disease must be promoted.

The most promising outcome for future study was time to MACE, with the observed HR indicating an RCT recruiting 528 patients could be definitive. All-cause mortality and DAOH were hypothesised to be expedient endpoints, but no signals of between-arm difference were observed. For all-cause mortality, this may reflect high degrees of frailty, and thus mortality risk for reasons not modifiable by treatment of heart disease in the population studied. DAOH was heavily left-skewed, limiting efforts to discern differences between arms. Over a longer follow-up period, it may have greater utility.

This study has limitations. As a pilot study, it was conducted in a single centre, potentially limiting generalisability. This would be addressed by a definitive multicentre study founded on these pilot results. Additionally, treatment allocation was not blinded. However, this is unlikely to have affected the assessment of key clinical outcomes, since these were determined by blinded mortality adjudication and by diagnosis and admission decisions made by independent clinical teams. Furthermore, the follow-up period of 12 months is shorter than commonly employed for trials using MACEs as an outcome, potentially limiting the ability to discriminate significant differences in this outcome. Lastly, aspects of the recruitment strategy and eligibility criteria potentially introduced selection bias, namely, that patients had to be able to consent and to undergo a CT scan. The former condition disadvantaged recruitment of ‘less unwell’ patients who were discharged before approach to participate, and the latter the ‘most unwell’ patients, who either could not lie flat—e.g., due to LVSD—or could not consent due to profound acute illness or cognitive impairment. Finally, it remains possible that SCA was not effective in the recruited population due to a high degree of frailty, meaning that no benefit was seen from therapies that have been proven to improve outcomes from HF and CAD in less frail cohorts.

Several strengths of this study merit emphasis. The eligibility criteria were broad, resulting in the recruitment of a sample that was highly representative of the real-world inpatient COPD population [30], despite the limitations mentioned above regarding recruitment. Furthermore, the diagnostic techniques that comprise SCA are, by intention, commonly available and inexpensive, and the management recommended for problems identified encompassed, in the main, inexpensive generic drug therapies, all of which have a strong evidence base supporting their use.

Further research directions are suggested by our results. Firstly, modification of the SCA to include recommending the use of SGLT2 inhibitors across a wider spectrum of patients with HF would enhance its efficacy. Recent evidence indicates these drugs reduce hospital admissions and mortality [31] in patients with HFpEF, which was found in 1 in 5 patients in this sample. Secondly, given that over 20% of patients had already experienced MACE at recruitment, a diagnostic intervention targeting CAD in patients with earlier-stage COPD may prove fruitful, although diagnostic rates may be lower. The early separation of admission-free survival curves seen in Figure 3B, if a replicable finding, further supports the application of SCA in less severe COPD, before severe COPD supervenes as the dominant cause of adverse outcomes.

## 5. Conclusions

In conclusion, in a pilot study, the use of a structured cardiac assessment identified new heart disease in 74% of patients receiving the intervention, with 56% started on new treatment. Heart failure was diagnosed in 36.8% of those undergoing SCA, and for patients with moderate–severe LVSD, it was associated with 57% one-year mortality. These findings strongly supporting the need for active case finding in all patients with hospitalised ECOPD. In the future, coordinators of COPD care should pivot towards systematic cardiovascular disease finding and treatment, basing pathways on CVD risk, rather than symptoms and signs. Future studies are necessary to definitively establish the optimum structured approach to this. These should consider using a CVD-specific endpoint as the primary outcome.

## Figures and Tables

**Table 1:** Baseline patient characteristics.

	SCA n = 57	Usual Care n = 58	*p* Value
Age, y	71.6 (6.34)	72.6 (6.54)	0.403
Sex, % female	59.6	56.9	0.851
Current smoking, % (n)	36.8 (21)	39.7 (23)	0.848
PYH, median (IQR)	45 (20)	50 (23)	0.441
BMI, kg/m^2^	25.9 (7.9)	24.8 (5.9)	0.431
Pre-admission FEV1 (% predicted)	50.2 (19.5)	48.0 (17.1)	0.532
eMRCD, median (IQR)	4 (4–5a)	4 (4–5a)	0.230
PEARL score, [23] median (IQR)	4 (1–5)	4 (1–5)	0.291
Patient reported ECOPD in past year, median (IQR)	3 (1–5)	3 (1.75–5)	0.941
ECOPD admissions in past year, median (IQR)	0 (0–1)	0 (0–1)	0.690
LTOT, %	10.5	15.5	0.581
Rockwood CFS, median (IQR)	5 (5–6)	5 (5–6)	0.657
Cardiovascular comorbidities, % (n)			
Moderate–severe LVSD	1.7 (1)	5.3 (3)	0.364
HF without moderate–severe LVSD	7.0 (4)	8.6 (5)	0.743
Right-sided HF	3.5 (2)	6.9 (4)	0.679
Myocardial infarction	14.0 (8)	13.8 (8)	1.000
Atrial fibrillation	7.0 (4)	8.6 (5)	1.000
Angina	17.5 (10)	8.6 (5)	0.177
Hypertension	50.9 (29)	43.1 (25)	0.457
High cholesterol	8.8 (5)	12.1 (7)	0.762
Stroke/transient ischaemic attack	19.3 (11)	19.0 (11)	1.000
Peripheral vascular disease	14.0 (8)	8.6 (5)	0.393
Diabetes	31.6 (18)	27.6 (16)	0.686
Chronic kidney disease	22.8 (13)	17.2 (10)	0.492
Age-adjusted Charlson comorbidity index, median (IQR)	5 (4–7)	5 (4–6)	0.181
COPD therapy, % (n)			
LABA + LAMA + ICS	87.8 (50)	75.9 (44)	0.181 *
LABA + LAMA	12.2 (7)	17.2 (10)	
LABA + ICS	0.0 (0)	3.4 (2)	
None	0.0 (0)	3.4 (2)	
Theophylline	8.8 (5)	3.4 (2)	0.272
Macrolide	28.1 (16)	22.4 (13)	0.525
Oral corticosteroid	7.0 (4)	12.1 (7)	0.528
Cardiovascular disease therapy, % (n)			
Aspirin	35.1 (20)	29.3 (17)	0.553
Dual antiplatelet therapy	3.5 (2)	1.7 (1)	0.618
Anticoagulation	7.0 (4)	12.0 (7)	0.528
Beta-blocker	19.3 (11)	22.4 (13)	0.819
ACE inhibitor/ARB	38.6 (22)	25.9 (15)	0.166
Statin	64.9 (37)	60.3 (35)	0.701
Other antihypertensive drug	29.8 (17)	24.1 (14)	0.534
Antidiabetic drug (inc. insulin)	15.8 (9)	15.5 (9)	1.000
MRA	1.8 (1)	1.7 (1)	1.000
Sacubitril–valsartan	0 (0)	0 (0)	-
SGLT2 inhibitor	1.8 (1)	0 (0)	0.496
NIV, % (n)	21.1 (12)	22.4 (13)	1.000
DECAF score, median (IQR)	2 (1–2)	2 (0–2)	0.880
Length of stay (days), median (IQR)	6 (3–8)	4 (3–9)	0.559
Died during admission, n	0	2	0.496

Mean and standard deviation unless stated. * Fisher’s exact test. Abbreviations: PYH—pack year history; IQR—interquartile range; BMI—body mass index; FEV1—forced expiratory volume in 1 s; eMRCD—extended MRC dyspnoea score; LTOT—long term oxygen therapy; CFS—clinical frailty score; LVSD—left ventricular systolic dysfunction; LABA—long-acting beta-agonist; LAMA—long-acting muscarinic antagonist; ICS—inhaled corticosteroid; ACE—angiotensin converting enzyme; ARB—angiotensin II receptor blocker; MRA—mineralocorticoid receptor antagonist; SGLT2—sodium-glucose co-transporter 2; NIV—non-invasive ventilation.

**Table 2:** New CVD diagnoses made during admission.

	SCA n = 57	UC n = 58
Heart failure		
Moderate–severe LVSD	5	2
HF without moderate–severe LVSD	13	4
Right-sided HF ^§^	8	2
Myocardial infarction	2	2
Atrial fibrillation	1	2
Mild coronary artery disease (CACS 1–100) *	11	0
Moderate–severe coronary artery disease (CACS > 100) *	34	0
Uncontrolled hypertension ^†^	14	0
Uncontrolled diabetes ^‡^	8	1

CACS—coronary artery calcium score. * Without pre-admission diagnosis of MI; ^§^ can coexist with LVSD; ^†^ BP above target range or antihypertensives increased during admission; ^‡^ HbA1C > 58 mmol/mol.

## Data Availability

The raw data supporting the conclusions of this article will be made available by the authors on request.

## Supplementary Materials

The following supporting information can be downloaded at https://www.mdpi.com/article/10.3390/biomedicines13030658/s1, S1: Trial protocol. S2: Management summaries. S3: CONSORT checklist. References [32,33,34,35,36,37,38,39,40,41,42,43,44,45,46,47,48,49,50,51,52] are cited in the supplementary materials.

## Abbreviations

The following abbreviations are used in this manuscript:

ACE	Angiotensin-converting enzyme
ARB	Angiotensin II receptor blocker
BMI	Body mass index
CACS	Coronary artery calcium score
CAD	Coronary artery disease
CFS	Clinical frailty score
COPD	Chronic obstructive pulmonary disease
CI	Confidence interval
CT	Computed tomography
CVD	Cardiovascular disease
DAOH	Days alive outside hospital
ECG	Electrocardiogram
ECOPD	Exacerbation of chronic obstructive pulmonary disease
ESC	European Society of Cardiology
eMRCD	Extended MRC dyspnoea score
FEV1	Forced expiratory volume in 1 s
FVC	Forced vital capacity
HbA_1C_	Haemoglobin A_1C_
HF	Heart failure
HR	Hazard ratio
ICS	Inhaled corticosteroid
IQR	Interquartile range
IRR	Incident rate ratio
LABA	Long-acting beta-agonist
LAMA	Long-acting muscarinic antagonist
LTOT	Long term oxygen therapy
LV	Left ventricle
LVEF	Left ventricular ejection fraction
LVSD	Left ventricular systolic dysfunction
MACE	Major adverse cardiovascular event
MI	Myocardial infarction
MRA	Mineralocorticoid receptor antagonist
NIV	Non-invasive ventilation
NT-pro-BNP	N-terminal pro-B-type natriuretic peptide
PEARL	PEARL prognostic score
PYH	Pack-year history
RCT	Randomised controlled trial
RR	Relative risk
SCA	Structured cardiac assessment
SD	Standard deviation
SGLT2	Sodium-glucose co-transporter 2
SGRQ-C	St. George’s Respiratory Questionnaire for COPD
UC	Usual care

## Appendix A

### Calculations of Sample Sizes Required to Achieve 80% Power in Future Trials

Calculation 1:

Primary outcome: death

Relative hazard = 0.903 (observed HR)

α (two tailed) = 0.05

β = 0.2

Proportion exposed = 0.5

Proportion unexposed = 0.5

Total events needed = 3016

Baseline event rate = 0.259 y-1

Median survival time (unexposed) = 2.676 y

Censoring rate = 0

Follow-up = 2 years

Sample size = 7754

Calculation 2:

Primary outcome: MACE

Relative hazard = 0.582 (observed HR)

α (two tailed) = 0.05

β = 0.2

Proportion exposed = 0.5

Proportion unexposed = 0.5

Total events needed = 107

Baseline event rate = 0.172 y-1

Median survival time (unexposed) = 4.03 y

Censoring rate = 0.165

Follow-up = 2 years

Sample size = 528
