# Supplementary material for: Structured Cardiac Assessment and Treatment Following Exacerbations of COPD (SCATECOPD): A Pilot Randomised Controlled Trial
Source: Biomedicines. 2025 Mar 7;13(3):658. doi: 10.3390/biomedicines13030658 (PMC11940601; doi:10.3390/biomedicines13030658)
Supplement: Supplementary file 1 [file biomedicines-13-00658-s001.zip › biomedicines-3476378-supplementary.pdf]

Supplementary Appendix

Contents	Page
Section S1: Trial protocol	2
Section S2: Management summaries	26
Section S3: CONSORT checklist	33

Section S1: Trial protocol

Title: Structured Cardiac Assessment and Treatment Following Exacerbations of COPD: a pilot randomised controlled trial

Short running title (acronym):

Structured cardiac assessment and treatment following exacerbations of COPD (SCATECOPD)

Key Trial Information

Chief Investigator:	Dr John Steer
Sponsor:	Northumbria Healthcare NHS Foundation Trust
Sponsor Reference Number:	NHCT0205
IRAS Number:	277817
REC Reference:	20/ES/0112
IRSCTN identifier:	ISRCTN26935612
NIHR CRN Portfolio ID:	47350
Funder:	Chiesi Limited & Northumbria Healthcare NHS Foundation Trust
Funding Reference Number:	Chiesi UK-CHI-2000241
Caldicott Approval Number:	C3502
Version number:	Version 1.5, 10th November 2021

Protocol developers

Dr John Steer	Consultant Respiratory Physician, Northumbria Healthcare NHS Foundation Trust and Newcastle University
Professor Stephen Bourke	Consultant Respiratory Physician, Northumbria Healthcare NHS Foundation Trust and Newcastle University
Dr David Ripley	Consultant Cardiologist, Northumbria Healthcare NHS Foundation Trust

Dr Keith Gray	Research Associate, Northumbria Healthcare NHS Foundation Trust. Statistician
Jo Gray	Associate Professor, Northumbria University. Health Economist

Contact Details

Northumbria Healthcare R&D department:	Tel:	08448118111
	Email:	ResearchAndDevelopment@northumbria-healthcare.nhs.uk
Chief Investigator:	Tel:	0191 2934351
	Email:	John.steer@nhct.nhs.uk

Signature Page

The undersigned confirm that the following protocol has been agreed and accepted and that the Chief Investigator agrees to conduct the trial in compliance with GCP guidelines, the Sponsor's (and any other relevant) SOPs, and other regulatory requirements as amended.

I agree to ensure that the confidential information contained in this document will not be used for any other purpose other than the evaluation or conduct of the clinical investigation without the prior written consent of the Sponsor.

I also confirm that I will make the findings of the trial publicly available through publication or other dissemination tools without any unnecessary delay and that an honest accurate and transparent account of the trial will be given; and that any discrepancies and serious breaches of GCP from the trial as planned in this protocol will be explained.

On behalf of the Trial Sponsor:

Signature: Date: ....../....../......

....

Name (please print):

....

Position: ....

Chief Investigator:

Signature: Date: 09/10/2020

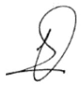

Name: (please print):

John Steer

Statistician:

Signature: Date: .09./10../.2020..

.. 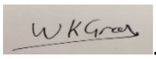...............................

Name: (please print):

William K Gray………………………………

Position: Senior Research Associate (Biostatistician)................

Roles and responsibilities

Chief Investigator	The Chief Investigator, John Steer, assumes primary responsibility for the design, conduct and reporting of the study.
Sponsor	The Sponsor, Northumbria Healthcare Foundation Trust, assumes overall responsibility for the initiation and management of the trial.
Protocol developers	The protocol developers, listed above, share responsibility for the trial protocol.
Statistician	The Statistician, Keith Gray, developed the statistical analysis plan. He will oversee final data analysis.
Funder	Chiesi Ltd have had no role in study design or protocol development and will have no role in the analysis or presentation of results.
Trial Steering Group	A Trial Steering Group will be formed with an independent Chair, and will meet at least every 12 months, and send timely reports to the Sponsor and Funder.
Trial Management Group	The Trial Management Group will meet regularly to ensure effective day-to-day running of the trial.

Trial Steering Group

Independent Chair
Patient Representative
Chief Investigator
Co-supervisor
Co-supervisor
Research Fellow
Statistician

APPROVALS & REGISTRATIONS TIMELINE

	Reference Number	*Date*
ISRCTN registration	ISRCTN26935612	29th October 2020
Caldicott approval	C3502	2nd November 2020
NIHR CRN support eligibility	47350	18th November 2020
Research & Ethics Committee favourable opinion	20/ES/0112	24th November 2020
HRA and HCRW approval	277817	14th December 2020
Local R&D approval	NHCT0205	14th December 2020

Contents

Supplementary Appendix 1
Supplementary figure .... **Error! Bookmark not defined.**
Trial protocol 2
Abbreviations 9
Protocol version history 10
Study synopsis 11
Lay summary 12
Scientific Rationale 13
Importance to patients and the NHS 15
Hypothesis 16
Objectives 16
Primary Objective 16
Secondary Objectives 16
Study Population 16
Inclusion criteria 16
Exclusion criteria 16
Target enrolment/sample size 16
Anticipated rate of enrolment: 16
Estimated study start date: 17
Estimated study completion date: 17
Study Design and Methods 17
Methods 17
Schedule of Activities 17

Economic Evaluation 20
Cardiovascular treatments 20
Covid19 precautions 20
Study Endpoints 21
Primary 21
Secondary 21
Statistical plan for data analysis 22
Adaptive design 22
Randomisation 22
Outcome measurements 22
Economic evaluation 22
Power calculation 23
Limitations 23
References 23
Management summaries 26
Sample Size calculations .... **Error! Bookmark not defined.**
Calculation 1: .... **Error! Bookmark not defined.**
Calculation 2: .... **Error! Bookmark not defined.**
CONSORT checklist 34

Abbreviations

ABG: Arterial Blood Gas 13
ACE: Angiotension Converting Enzyme 9
AF: Atrial Fibrillation 9
BP: Blood Pressure 14
BSE: British Society of Echocardiography 14
CVD: Cardiovascular Disease 9
DMEC: Data Monitoring and Ethics Committee 16
ECG: Electrocardiograph 13
HFpEF: Heart Failure with Preserved Ejection Fraction 9
HFrEF: Heart Failure with Reduced Ejection Fraction 9
LV: Left Ventricle or Left Ventricular 9
NHS: National Health Service 10
NICE: National Institute for Health and Care Excellence 10
NYHA: New York Heart Association 13
PEARL: Previous Admissions, Extended MRC Dyspnoea Score, Age, Right and Left Ventricular Failure Score 16
QALY: Quality Adjusted Life Years 7, 12
SGRQ: St. George's Respiratory Questionnaire 13

Protocol version history

Version	Date	Authors	Changes
1.0	05/10/2020	John Steer, Stephen Bourke, David Ripley, Joe Kibbler, Jo Gray, Keith Gray	N/A
1.1	14/10/2020	John Steer, Stephen Bourke, David Ripley, Joe Kibbler, Jo Gray, Keith Gray	Addition of need for clinical stability at 90-day review (P.14). Correction of name of agent used for contract echocardiography (P.14).
1.2	11/11/2020	John Steer, Stephen Bourke, David Ripley, Joe Kibbler, Jo Gray, Keith Gray	Clarification of process for capturing healthcare resource use (P.13). Addition of information about timing of study procedures (P.14). Addition of information to be collected about cardiovascular treatments (P.15).
1.3	17/12/2020	John Steer, Stephen Bourke, David Ripley, Joe Kibbler, Jo Gray, Keith Gray	Added Approvals & Registrations Timeline (P.3).
1.4	26/05/2021	John Steer, Stephen Bourke, David Ripley, Joe Kibbler, Jo Gray, Keith Gray	Added amendments regarding not repeating recent CT coronary artery calcium scoring (P.16) and clinical assessment and definition of clinical stability (P. 15)
1.5	10/11/2021	John Steer, Stephen Bourke, David Ripley, Joe Kibbler, Jo Gray, Keith Gray	Added amendment regarding (P.15) cut-off for 90-day follow-up visits delayed by exacerbations

Study synopsis

**Title**	**Structured Cardiac Assessment and Treatment Following Exacerbations of COPD: a pilot randomised controlled trial (SCATECOPD)**
**Sponsor**	Northumbria Healthcare NHS Foundation Trust
**Design**	Pilot randomised controlled trial
**Population**	Patients admitted to hospital with an exacerbation of COPD
**Sample size**	120
**Study duration**	12 months
**Planned Trial period**	Nov 2020 to Feb 2023
**Primary Objective**	To assess the effect of comprehensive cardiovascular assessment and treatment on the primary endpoint to enable powering of a definitive multicentre RCT
**Secondary Objectives**	1. Report the rates of CVD; specifically, the rates of undiagnosed or undertreated CVD.<br>2. Examine the utility of the primary outcome compared to readmissions, mortality and quality of life.<br>3. Compare the observed treatment effect between primary and secondary outcomes<br>4. Examine the relationship between changes in cardiac function (in the intervention group) from baseline to 90 days and E(COPD) severity, comorbid CVD, and outcome.<br>5. Assess the feasibility of collecting service-use data for an economic evaluation of the intervention in a future RCT<br>6. Report differences in health costs and estimated quality adjusted life years (QALY) between the 2 study arms.

Lay summary

Chronic obstructive pulmonary disease (COPD) is a common lung disease which can flare up and need admission to hospital. Patients with COPD often have heart disease, which worsens their symptoms and increase the chances of death and hospital admission. In the short period after a flare up of COPD, patients are also at a higher risk of heart attacks and irregular heart rhythms, which cause many of deaths and hospital readmissions experienced. Unfortunately, heart disease is often not recognised or not treated adequately in patients with COPD.

We will test whether carefully finding and treating heart disease in patients admitted to hospital with COPD exacerbation is beneficial. 120 patients will take part in this study. 60 patients will be randomly allocated to 'intervention group' and have detailed tests to identify and then treat heart disease. We will compare their outcomes to 60 patients who do not have this assessment.

In all 120 patients, we will record routine clinical information, simple questionnaires and breathing tests. These tests will be repeated 3 and 12 months later. The 60 patients in the intervention group will have detailed heart tests (blood tests, heart scans, and heart monitors). If we find a heart problem we will start treatment. We will compare the two groups to see if patients in the intervention group spend more time living at home during the year after they start the study.

Scientific Rationale

This project addresses a healthcare priority. Exacerbations of COPD (ECOPD) are a frequent cause of non-elective hospital admission and are associated with high rates of hospital readmission; 43% of patients were readmitted within 90 days following ECOPD according to the most recent national COPD audit report. Mortality following hospital admission for ECOPD is common, with rates of up to 50% in the most unwell patients during the 12 months following admission. Cardiovascular disease (CVD) is common in COPD and is associated with worse quality of life, and higher mortality and readmission rates [1]. The risks of myocardial infarction and hospital admission for atrial fibrillation (AF) are substantially higher than baseline risk during the short period after hospital admission for ECOPD [2,3].

Despite these known risks, CVD is often undertreated and / or not recognised. Three months after ECOPD requiring hospital admission, a quarter of patients with no past history of cardiac disease have left ventricular (LV) dysfunction and 44% have right heart impairment [4]. In patients with COPD attending pulmonary rehabilitation, 16.5% of patients had impaired LV function (previously undiagnosed in 36%) and 20% had pulmonary hypertension (undiagnosed in two thirds) [5] There are fewer studies investigating rates of CVD and cardiac dysfunction in patients at the time of ECOPD. Many studies rely on ICD coding diagnoses to define CVD and few clarify the presence of undiagnosed CVD. Marcun et al [6,7] performed echocardiography on 127 patients admitted with ECOPD. An abnormality of left ventricular function was identified in 55% of patients and prevalence of heart failure with reduced (HFrEF) and preserved (HFpEF) ejection fraction was 9% and 19% respectively. A subsequent study of 154 patients from the same group [8] reported 10% of patients had HFrEF and diastolic dysfunction was present in over 50%. These studies did not report on treatment or whether abnormalities had been previously recognised. AF has been reported to be present in between 17% and 35%, [9,10] with rates higher in more severe ECOPD, but the proportion of patients with new AF is unknown.

Even if CVD is diagnosed, it is frequently undertreated. Beta blocker and ACE-inhibitor prescriptions are lower in patients with COPD than those without COPD, even when there is evidence of a clear prognostic benefit from their use [11–13]. Following myocardial infarction, patients with COPD are less likely to receive treatment with aspirin and statins than patients without COPD [14].

In addition to the cardiovascular benefits of treating CVD appropriately, cardiovascular-specific therapies can improve respiratory-specific outcomes in COPD. The CHAMPION [15] study showed that, in the subgroup of patients with COPD, adjusting heart failure drugs using data from an implantable pulmonary artery pressure monitoring device reduced hospitalisations to both heart failure and respiratory disease. In severely unwell

patients with ECOPD requiring critical care, unrecognised HFrEF was present in 41%, and these patients had better clinical outcomes suggesting, but not proving, that identifying and treating CVD could improve outcome [16]. Observational studies have shown that patients with COPD receiving treatment with beta-blockers have lower mortality rates [17]. A recent RCT [18] giving beta-blockers to patients with COPD without an indication for beta-blocker therapy did not show a benefit compared to placebo; suggesting that the previously identified improved outcomes from betablocker therapy are due to the better treatment of previously unrecognised or undertreated CVD.

Pulmonary hypertension is a marker of severe COPD and independently associated with future ECOPD [19]. Pulmonary artery pressures are often elevated at the time of ECOPD and improve following ECOPD recovery [20]. It is unclear how changes in pulmonary artery pressures relate to COPD severity, exacerbation severity and CVD, and whether acute changes in pulmonary artery pressure have an impact on patients' longer term outcome. This project will help us understand more about these relationships.

We propose a randomised pilot study to investigate the impact of a comprehensive cardiac assessment (and standardised treatment of CVD) on days spent alive outside of hospital during 12 months follow up. This patient centred primary outcome measure captures the number and duration of hospital admissions as well as mortality and, whilst it has been used in cardiovascular studies, [21] it is a novel outcome measure in COPD. Data from this pilot study will hopefully lead to a larger, multicentre randomised controlled trial (not part of this proposal). This would be the first study to our knowledge to investigate the impact of treating CVD on outcome in ECOPD and the first study to use this novel patient outcome.

The aims of this project are congruent with many national priorities and recommendations. *Multimorbidity: a priority for global health research*, a report from The Academy of Medical Sciences (April 2018), listed six research priorities. Our proposal addresses Research Priority 6 which highlights the need for research in to integrated healthcare strategies which "improve clinical outcomes, patient-centred outcomes, and the cost-effectiveness of care". The NHS Outcomes Framework identifies: reducing readmissions within 30 days of hospital discharge (Indicator 3b); and reducing mortality from respiratory disease in under 75s (Indicator 1.2) as key markers of healthcare performance. The NICE Multimorbidity Guideline (NG56) recommends that research in to the holistic assessment of multimorbidity should have clear identification of the target population, careful piloting, well planned interventions, and be directed at outcomes relevant to patients (such as quality of life, hospital admission and mortality); the present proposal meets all of these criteria.

Importance to patients and the NHS

The 2017 National Asthma and COPD Audit Programme (NACAP) COPD Advisory Group highlighted, as a key message, the need for a holistic approach to care focusing on multimorbidity [22]. The COPD Advisory Group has patient representation at its heart and can be relied upon to reflect the priorities of both clinicians and patients. This research proposal can yield the following results:

1. Show that a structured cardiovascular assessment and treatment improves outcome. The reduction in readmissions, bed days and mortality could be substantial.
2. Enable a definitive RCT to be designed. Our results will help refine the methodology and enable a power calculation. The proposed RCT could help embed our structured cardiac assessment in the care of patients across the NHS.
3. Provide data to inform a full health economic analysis in the planned subsequent RCT. The costs of hospital admissions are substantial compared to the intervention under investigation.
4. Highlight the burden of CVD, particularly when undiagnosed or under-treated.

These results can then benefit patients by:

1. Highlighting to clinicians the significance of cardiovascular disease for patients hospitalised with ECOPD. An increased awareness will benefit patients with (E)COPD by reducing the diagnostic and treatment gap.
2. Showing, to commissioners and healthcare organisations, the benefit that can be yielded from ensuring sufficient access to cardiovascular diagnostics.
3. Establishing the utility of a novel outcome measure for ECOPD.
4. Improvements in in-hospital mortality rates in ECOPD have been seen over recent years due to improved service delivery; no new treatments have become available. Better diagnosis and management of cardiovascular disease could substantially improve patient outcomes and reduce health resource use.

Hypothesis

A comprehensive, structured cardiovascular assessment, with treatment of problems identified, increases the time patients spend alive outside of hospital following hospital admission for a COPD exacerbation.

Objectives

Primary Objective

To assess the effect of comprehensive cardiovascular assessment and treatment on the primary endpoint to enable powering of a definitive multicentre RCT.

Secondary Objectives

1. Report the rates of CVD; specifically, the rates of undiagnosed or undertreated CVD.
2. Examine the utility of the primary outcome compared to readmissions, mortality and quality of life.
3. Examine the relationship between changes in cardiac function (in the intervention group) from baseline to 90 days, and (E)COPD severity and comorbid CVD.
4. Assess the feasibility of collecting service-use data for an economic evaluation of the intervention in a future RCT
5. Report differences in health costs and estimated quality adjusted life years (QALY) between the 2 study arms.

Study Population

Patients hospitalised to Northumbria Specialist Emergency Care Hospital with an exacerbation of spirometry confirmed COPD.

Inclusion criteria

- Age >35 years
- Current / former smoker & smoking burden >10 pack years
- Clinical diagnosis of COPD, supported by previous obstructive spirometry
- Admission to hospital with the primary cause being an exacerbation of COPD

Exclusion criteria

- Reason for admission not ECOPD in view of attending clinical team.
- Unable to provide informed consent
- Any non-COPD condition likely to limit survival to less than 12 months
- Contra-indication to cardiac CT
- Pregnancy or breastfeeding

Target enrolment/sample size:

120

Anticipated rate of enrolment:

7-8 per month

Estimated study start date:

First patient first visit: 28th November 2020

Estimated study completion date:

Last patient last visit: 28th February 2023

Study Design and Methods

Methods

A randomised (1:1) pilot study of 120 consecutive consenting patients. The intervention group will undergo a comprehensive cardiac assessment and standardised treatment protocols will be followed to treat identified CVD. Patients will be followed for 12 months to gather outcome data. CVD is defined as hypertension, ischaemic heart disease (IHD), HFPEF, HFREF, atrial or ventricular arrhythmia, moderate-severe valvular heart disease or cor pulmonale.

Schedule of Activities

All patients will have the following assessments (conducted face to face unless specified):

	Baseline	90 (+/- 10) days†	6 months (+/- 10 days) - telephone	9 months (+/- 10 days) - telephone	12 months (+/- 10 days)
Demographics, comorbidity	X	X			X
Medications	X	X	X	X	X
eMRCD	X	X			X
Spirometry & inspiratory capacity	X	X			
NYHA class	X	X			
Rockwood clinical frailty scale	X				
COPD assessment*	X				
Bedside observations$^{\alpha}$	X				
ABG	X				

ECG	X				
Laboratory tests[β]	X				
4m gait speed	X	X			X
SGRQ-C	X	X			X
EQ-5D-5L	X	X			X
Exacerbation frequency	X	X	X	X	X
Hospital admissions / ED attendances		X			X
Primary & community NHS care visits[π]		X	X	X	X
Mortality					X
Adverse Cardiovascular Events ‡		X			X

† - at clinical stability, defined as > 6 weeks after completion of primary treatment course for COPD exacerbation; * - includes; BMI, previous spirometry, previous exacerbation / hospital admission / NIV episodes, α- components of NEWS2 scale; π – health service utilisation form; β – full blood count, urea & electrolytes, liver function tests, glucose, albumin, lactate, CRP; ‡ nonfatal stroke or myocardial infarction, and cardiovascular death

In addition to face to face assessments, all patients will be contacted by telephone at 6 and 9 months to complete an assessment of healthcare resource use. In hospital and at 3, 6 and 9 months following hospital discharge, patients will be provided with a health service utilisation form and asked to use this to record all contact with community NHS services. This data will be reviewed with the patient at the face to face (3 and 12 months) and telephone (6 and 9 months) assessments. Data regarding frequency of COPD exacerbations will be collected at these time points; severe exacerbations (requiring admission) will be captured from health records, moderate exacerbations (requiring treatment with antibiotics and/or steroids) will be self-reported.

Clinical stability is required at the 90-day review for accurate assessment of spirometry and, for those randomised to structured cardiovascular assessment, for echocardiography. The 90-day review appointment will therefore be delayed, if necessary, until participants are in a period of clinical stability, which is defined as more than 4 weeks after completion of a course of steroids and/or antibiotics for a diagnosed COPD exacerbation. The research fellow may clinically assess participants to determine if a COPD exacerbation should be diagnosed if a patient is unwell around the time of review. To avoid excessive delay in cases

of recurrent exacerbation, 90 day review will be conducted after the delay has reached 8 weeks, regardless of the recency of exacerbation.

Those randomised to the structured cardiovascular assessment will also undergo:

	Baseline	90 days
ECG	X	X
Echocardiogram	X	X
Laboratory investigations$^{\beta}$	X	X
24 hour cardiac monitor	X	
CT coronary artery calcification score and CT chest	X$^{\gamma}$	
24 hour BP monitor$^{◎}$	X	

β – troponin T, NT pro-BNP, fibrinogen, cholesterol profile (admission only), HbA1c (admission only);. γ - performed at any point during index hospital admission (or within 7 days of admission); ◎ - performed in patients hypertensive during initial hospital stay, without a prior hypertension diagnosis

Echocardiography will be performed by a trained clinician or British Society of Echocardiography (BSE) trained physiologist with oversight by an independent consultant imaging cardiologist. If image quality is insufficient for accurate assessment of ventricular function, contrast echocardiography using SonoVue contrast agent will be performed during inpatient admission.

CT Coronary artery calcification score will be performed according to standard protocol by a trained consultant Cardiologist. Reporting will be performed blinded to the presence of known CVD and knowledge of severity of COPD and ECOPD. Assessment of whether CVD is treated adequately will be made with reference to (inter)national guidance. CT chest will be performed without intravenous contrast according to standard local protocol; emphysema severity and airway wall thickening quantified using commercially available software. Slots for CT scanning for of the study are available at the start of the morning list so it is anticipated that all patients will be able to have a CT scan before being discharged. If this is not possible patients can have their CT scan on an urgent outpatient basis, within 7 days of discharge. This will avoid discharge being delayed for study investigations. If patients have had a CT coronary artery calcium score calculated within 6 months prior to recruitment, this test will not be repeated as the score is unlikely to have changed significantly in this time.

Patients who require 24 hour blood pressure monitoring will either have this test during their inpatient stay or it will be done soon after discharge (a member of the research team will fit the monitor at the patient's place of residence and collect it the following day).

Economic Evaluation

A prospective economic evaluation will be rehearsed to develop and refine methods for a subsequent definitive trial. The main focus will be on how to accurately identify, quantify and value the additional costs of delivering the intervention and the potential resource implications versus usual care. The costing approach will incorporate an NHS perspective (Secondary, Primary & Community care costs), which will help to detect cost-shifting between NHS care sectors. Resources utilised in the intervention group will be identified in terms of CVD investigations, medications and staff time. Subsequent resource utilisation during follow-up will be captured using a number of NHS databases and a patient resource utilisation pro-forma. In-hospital data including A&E attendances and hospital admissions will be collected through case note review and use of hospital coding services. Primary care attendances including GP visits and Nurse visits will be obtained using a patient resource utilisation pro-forma. This will be assessed retrospectively at the four follow up periods (3, 6, 9 and 12 months). This will facilitate the development of a reliable and valid tool to capture resource use. Data on use of services will be combined with appropriate unit cost to produce a cost per trial participant. These will be sourced from a combination of local costings and national databases [23,24].

Cardiovascular treatments

For any cardiovascular disease identified, treatment will be initiated by the usual care team in keeping with current local (Northumbria Healthcare NHS Foundation Trust) or (inter)national guidelines. No novel treatment regimens are being examined in this study. Communication with the patient's primary care team will be clear and optimisation of treatment will be performed by the GP, in keeping with (inter)national guidance. In order to facilitate optimum communication between secondary care, primary care and the patient, management summaries (summarising the relevant current guidance reflecting best practice) will be written by the supervisory team with the input of local General Practitioners and an expert patient representative. Treatments given to patients will be recorded at follow up visits as part of the assessment of the rate of undertreated cardiovascular disease (a secondary study outcome). Treatment protocols will be accessible online in primary care and published on the trust website. Treatment of patients in usual care arm will be at the discretion of the treating clinicians.

Covid19 precautions

All research activity will adhere to the strict Northumbria Healthcare NHS Foundation Trust infection control procedures active at the time. Additionally, patients will be contacted prior to attending hospital to ensure they are not showing symptoms of Covid19 infection. We will reschedule this appointment if patients are symptomatic. Additional procedures to minimise the risk of transmitting Covid19 include: social distancing will be adopted during all face-to face assessments; members of the research team will wear the necessary PPE as advised by Public Health England and the Trust infection control polices during the

assessments and investigations; we will aim to minimise patients' exposure to healthcare professionals during their assessments and aim to limit the waiting times within hospital; and study participants will use a separate entrance to the research and development centre, minimising exposure to healthcare professionals, patients and hospital visitors.

Study Endpoints

Key outcome measures of interest include readmission and mortality. Our primary outcome measure captures both readmission and death, reflects the number and duration of admissions, and places a higher weighting on mortality (particularly if soon after randomisation). To our knowledge this has not been used in a previous trial involving patients with COPD. Secondary outcomes are included to examine the utility of our novel primary outcome, and to gather important descriptive data to enable powering of the subsequent definitive RCT. Readmission, mortality and length of hospital stay will be collected from hospital health records, using Patient Administration System (PAS).

Primary

1. The number of days spent alive outside of a hospital environment during 12 months post hospital discharge

Secondary

1. Time to readmission or death following hospital admission for ECOPD
2. All-cause readmission rates at 90 days and 12 months post discharge
3. All-cause mortality rates at 90 days and 12 months post discharge
4. COPD exacerbation rates, from health records and self-reported, at 90 days and 12 months.
5. Rates of adverse cardiovascular events* at 90 days and 12 months post discharge
6. Rate of new diagnosis of cardiovascular disease at 90 days and 12 months
7. Rate of undertreated cardiovascular disease at baseline, 90 days and 12 months
8. Change in 4 metre gait speed at 90 days and 12 months, compared to baseline
9. Mean change in quality of life measured by St. Georges' Respiratory Questionnaire over 12 months.
10. Health costs and estimated Quality Adjusted Life Years (QALY), measured by health records and patient-completed resource utilisation proforma, at 12 months

In the intervention arm we will also report as secondary outcomes:

11. Changes in right heart function† between baseline and 90 days
12. Relationship between changes in right heart function† and ECOPD severity measured using DECAF score
13. Relationship between changes in right heart function† and comorbid CVD
14. Relationship between right heart function† and COPD severity at baseline.
15. The associations between the primary outcome and right heart function at baseline†.

* nonfatal stroke or myocardial infarction, and cardiovascular death; † Estimated pulmonary artery systolic pressure (PASP) and tricuspid annular plane systolic excursion (TAPSE) measured by echocardiography.

Statistical plan for data analysis

Adaptive design

Within the present study, an interim statistical analysis will be undertaken after the first 80 subjects have completed follow up. If a realistic extension to this study could achieve clinically and statistically meaningful conclusions regarding the primary outcome we will extend recruitment.

Randomisation

Patients will be randomly assigned 1:1 to the intervention or usual care. Independent stratified randomisation (via sealedenvelopes.com) using the PEARL score [25] (low, medium and high risk) and the presence of known CVD pre-hospital admission will be performed.

Outcome measurements

Changes in the primary outcome between the intervention and usual care groups will be assessed using Student's t-test or Mann-Whitney U test. Time to first event (readmission or death) will be assessed using a Cox proportional hazards regression model. Changes in echocardiographic measurements between two time points will be assessed using paired Student's t-test or Wilcoxon signed-rank test. The relationship between undiagnosed / undertreated cardiac disease and outcome will be examined using logistic regression. In those surviving to the first follow up assessment, correlations between changes in right heart function and measures of COPD severity and exacerbation severity will be examined using bivariate comparisons appropriate to variable distribution. Mean change in QoL will be calculated by area under the curve per unit time; Student's t-test or Mann Whitney U will be used to compare this value between study arms.

Economic evaluation

The methods to estimate an incremental cost-effectiveness ratio for the intervention versus usual care in terms of Quality Adjusted Life Years will be rehearsed (using EQ-5D-5L administered at baseline, 90 weeks and 12 months post discharge). In particular, issues relevant for sensitivity analysis will be explored to help understand how best to deal with statistical imprecision and other uncertainties in the full trial. For example, data will be bootstrapped to account for the expected skewness evident in economic cost data. The data collected as part of this feasibility study could be used to inform any subsequent pre-trial modelling.

Power calculation

This is a pilot study. The lack of published data regarding both the prevalence of undiagnosed cardiovascular disease in patients hospitalised with ECOPD, and the impact of cardiovascular assessment (and treatment) on our primary outcome means a power calculation is not possible. We have previously successfully recruited 118 patients hospitalised with mild ECOPD over 18 months to a RCT of hospital at home in ECOPD. This recruited from a smaller population than the present proposal (~50% of total population) and involved a complex intervention.

Readmission and mortality rates are high following ECOPD; based on our pilot data, we are optimistic that our chosen sample size will be deliverable and show a treatment effect that will enable a definitive trial to be powered.

Limitations

This study examines the effect of a novel intervention on a novel COPD outcome. The aim is to use the results to power a definitive RCT, but a lack of a treatment effect may mean a definitive trial is superfluous. Adequate echocardiographic images to make reliable measurements can be challenging in patients with COPD, particularly during an exacerbation when they are more breathless. Whilst a reduction in the amount of echocardiographic data available may reduce the ability to diagnose patients with HFpEF, HFrEF or valvular heart disease, this information will be vital in order to adequately power a definitive trial.

References

1. MacDonald, M.I; Shafuddin, E.; King, P.T.; Chang, C.L.; Bardin, P.G.; Hancox, R.J. Cardiac dysfunction during exacerbations of chronic obstructive pulmonary disease. *Lancet Respir. Med.* **2016**, *4*, 138–148.
2. Donaldson, G.C.; Hurst, J.R.; Smith, C.J.; Hubbard, R.B.; Wedzicha, J.A. Increased risk of myocardial infarction and stroke following exacerbation of COPD. *Chest* **2010**, *137*, 1091–1097.
3. Atsushi, H.; Tadahiro, G.; Yuichi J, S.; Kamal, F.M.; Carlos A, C.; Kohei, H. Acute Exacerbation of Chronic Obstructive Pulmonary Disease and Subsequent Risk of Emergency Department Visits and Hospitalizations for Atrial Fibrillation. *Circ. Arrhythm Electrophysiol.* **2018**, *11*, e006322.
4. Freixa, X.; Portillo, K.; Paré; C; Garcia-Aymerich, J.; Gomez, F.P.; Benet, M.; Roca, J.; Farrero, E.; Ferrer, J.; Fernandez-Palomeque, C.; Anto, J.M. Echocardiographic abnormalities in patients with COPD at their first hospital admission. *Eur. Respir. J.* **2013**, *41*, 784–791.
5. Houben-Wilke, S.; Spruit, M.A.; Uszko-Lencer, N.H.; Otkinska, G.; Vanfleteren, L.E.; Jones, P.W.; Wouters, E.F.; Franssen, F.M. Echocardiographic abnormalities and their impact on health status in patients with COPD referred for pulmonary rehabilitation. *Respirology* **2017**, *22*, 928–934.

6. Marcun, R.; Sustic, A.; Brguljan, P.M.; Kadivec, S.; Farkas, J.; Kosnik, M.; Coats, A.J.S.; Anker, S.D.; Lainscak, M. Cardiac biomarkers predict outcome after hospitalisation for an acute exacerbation of chronic obstructive pulmonary disease. *Int. J. Cardiol.* **2012**, *161*, 156–159.
7. Marcun, R.; Stankovic, I.; Vidakovic, R.; Farkas, J.; Kadivec, S.; Putnikovic, B.; Ilic, I.; Neskovic, A.N.; Lainscak, M. Prognostic implications of heart failure with preserved ejection fraction in patients with an exacerbation of chronic obstructive pulmonary disease. *Intern. Emerg. Med.* **2015**, *11*, 519–527.
8. Stankovic, I.; Marcun, R.; Janicijevic, A.; Farkas, J.; Kadivec, S.; Ilic, I.; Neskovic, A.N.; Lainscak, M. Echocardiographic predictors of outcome in patients with chronic obstructive pulmonary disease. *J. Clin. Ultrasound* **2016**, *45*, 211–221.
9. Echevarria, C.; Steer, J.; Heslop-Marshall, K.; Stenton, S.; Hickey, P.; Hughes, R.; Wijesinghe, M.; Harrison, R.N.; Steen, N.; Simpson, A.; et al. Validation of the DECAF score to predict hospital mortality in acute exacerbations of COPD. *Thorax* **2016**, *71*, 133–140.
10. Terzano, C.; Romani, S.; Conti, V.; Paone, G.; Oriolo, F.; Vitarelli, A. Atrial fibrillation in the acute, hypercapnic exacerbations of COPD. *Eur. Rev. Med. Pharmacol. Sci.* **2014**, *18*, 2908–2917.
11. Lipworth, B.; Skinner, D.; Devereux, G.; Thomas, V.; Jie, J.L.Z.; Martin, J.; Carter, V.; Price, D.B. Underuse of β-blockers in heart failure and chronic obstructive pulmonary disease. *Heart* **2016**, *102*, 1909–1914.
12. Mentz, R.J.; Schmidt, P.H.; Kwasny, M.J.; Ambrosy, A.P.; O'Connor, C.M.; Konstam, M.A.; Zannad, F.; Maggioni, A.P.; Swedberg, K.; Gheorghiade, M. The Impact of Chronic Obstructive Pulmonary Disease in Patients Hospitalized for Worsening Heart Failure with Reduced Ejection Fraction: An Analysis of the EVEREST Trial. *J. Card. Fail.* **2012**, *18*, 515–523.
13. Egred, M.; Shaw, S.; Mohammad, B.; Waitt, P.; Rodrigues, E. Under-use of beta-blockers in patients with ischaemic heart disease and concomitant chronic obstructive pulmonary disease. *Qjm: Int. J. Med.* **2005**, *98*, 493–497.
14. Rasmussen, D.B.; Bodtger, U.; Lamberts, M.; Nicolaisen, S.K.; Sessa, M.; Capuano, A.; Torp-Pedersen, C.; Gislason, G.; Lange, P.; Jensen, M.T. Beta-blocker, aspirin, and statin usage after first-time myocardial infarction in patients with chronic obstructive pulmonary disease: a nationwide analysis from 1995 to 2015 in Denmark. *Eur. Heart J. Qual. Care Clin. Outcomes* **2019**, *6*, 23–31. Available online: https://academic.oup.com/ehjqcco/advance-article/doi/10.1093/ehjqcco/qcy063/5273489 (accessed on 15 November 2019).
15. Krahnke, J.S.; Abraham, W.T.; Adamson, P.B.; Bourge, R.C.; Bauman, J.; Ginn, G.; Martinez, F.J.; Criner, G.J. Heart Failure and Respiratory Hospitalizations Are Reduced in Patients with Heart Failure and Chronic Obstructive Pulmonary Disease with the Use of an Implantable Pulmonary Artery Pressure Monitoring Device. *J Card Fail.* **2015**, *21*, 240–249.
16. Matamis, D.; Tsagourias, M.; Papathanasiou, A.; Sineffaki, H.; Lepida, D.; Galiatsou, E.; Nakos, G. Targeting occult heart failure in intensive care unit patients with acute chronic obstructive pulmonary disease exacerbation: Effect on outcome and quality of life. *J. Crit. Care* **2014**, *29*, 315.e7–315.e14.

17. Dransfield, M.T.; Rowe, S.M.; Johnson, J.E.; Bailey, W.C.; Gerald, L.B. Use of beta blockers and the risk of death in hospitalised patients with acute exacerbations of COPD. *Thorax* **2008**, *63*, 301–305.
18. Dransfield, M.T.; Voelker, H.; Bhatt, S.P.; Brenner, K.; Casaburi, R.; Come, C.E.; Cooper, J.A.D.; Criner, G.J.; Curtis, J.L.; Han, M.K.; et al. Metoprolol for the Prevention of Acute Exacerbations of COPD. *New Engl. J. Med.* **2019**, *381*, 2304–2314.
19. Wells, J.M.; Washko, G.R.; Han, M.K.; Abbas, N.; Nath, H.; Mamary, A.J.; Regan, E.; Bailey, W.C.; Martinez, F.J.; Westfall, E.; et al. Pulmonary Arterial Enlargement and Acute Exacerbations of COPD. *New Engl. J. Med.* **2012**, *367*, 913–921.
20. Ozben, B.; Eryuksel, E.; Tanrikulu, A.M.; Papila, N.; Ozyigit, T.; Celikel, T.; Basaran, Y. Acute Exacerbation Impairs Right Ventricular Function in COPD Patients. *Hellenic J. Cardiol.* **2015**, *56*, 324–331.
21. Fanaroff Alexander C., Cyr Derek, Neely Megan L., Bakal Jeffery, White Harvey D., Fox Keith A.A., et al. Days Alive and Out of Hospital: Exploring a Patient-Centered, Pragmatic Outcome in a Clinical Trial of Patients With Acute Coronary Syndromes. *Circ Cardiovasc. Qual. Outcomes* **2018**, *11*, e004755.
22. Hurst, J.; Stone, R.A.; McMillan, V.; National Asthma Chronic Obstructive Pulmonary Disease Audit Programme (NACAP). Outcomes of patients included in the 2017 COPD clinical audit (patients with COPD exacerbations discharged from acute hospitals in England and Wales between February and September 2017). London: RCP; 2019 May. (National Asthma and Chronic Obstructive Pulmonary Disease Audit Programme (NACAP)).
23. Department for Health and Social Care (DHSC). National Cost Collection for the NHS. London: DHSC; 2019. Available online: https://www.england.nhs.uk/national-cost-collection/#ncc1819 (accessed on 27 August 2020)
24. Curtis, L.BA. Unit Costs of Health and Social Care 2019. Cantebury: PSSRU; University of Kent; 2019. Available online: https://www.pssru.ac.uk/project-pages/unit-costs/unit-costs2019/ (accessed on 27 August 2020)/
25. Echevarria, C.; Steer, J.; Heslop-Marshall, K.; Stenton, S.C.; Hickey, P.M.; Hughes, R.; Wijesinghe, M.; Harrison, R.N.; Steen, N.; Simpson, A.J.; et al. The PEARL score predicts 90-day readmission or death after hospitalisation for acute exacerbation of COPD. *Thorax* **2017**, *72*, 686–693.

Section S2: Management summaries

SCATECOPD Management summary: heart failure

Definitions

The following practical definitions reflect implementation of evidence and international guidelines by local cardiology services:

1. Heart failure with moderate-severe LV systolic impairment
   - LVEF < 45% at transthoracic echocardiography (echo)
2. Heart failure without moderate-severe LV systolic impairment
   - LVEF ≥ 45% with echo report of LV diastolic dysfunction
3. Right sided heart failure:
   - Echo evidence of RV impairment in patient with peripheral oedema

Echo should be performed as early as possible during admission. If NIV used, wait until weaned to periods of ≥ 4 hours off NIV

Treatment goals

1. In patients with moderate-severe LV impairment, establishment of maximum tolerated dose of beta blocker and ACE-inhibitor, +/- spironolactone, with referral to heart failure team for supervision of this
2. In all other patients with heart failure, euvolaemia and control of hypertension, AF and CAD if present

Acting on echo reports

1. In patients with moderate-severe LV systolic impairment refer to heart failure service for ongoing supervision of the below:

a) Start bisoprolol 1.25mg provided heart rate (HR) above 65 bpm and ECG excludes 2nd/3rd degree AV block

- If already on bisoprolol increase dose if HR > 65 bpm
- If on carvedilol, this can be continued/increased depending on HR
- If on a different beta blocker, switch to bisoprolol at equivalent dose

Titrate beta blocker dose every 2 weeks initially, aiming for HR < 70 bpm

b) At the same time, introduce ramipril 1.25mg OD provided not hypotensive or hyperkalaemic, or on ARB

- If cough/other insensitivity to ACE-inhibitor, introduce losartan 12.5mg OD

Titrate ACE-inhibitor/ARB dose every 2 weeks initially, aiming for normal BP and stable creatinine

c) If clinically hypervolaemic and not on diuretics, introduce furosemide 40mg OD

- If already on furosemide increase dose by 40mg/d

d) When beta blocker and ACE inhibitor doses stabilised, introduce spironolactone 25mg OD if LVEF < 35%.

2. For all other patients with heart failure:

If admission CXR shows pulmonary congestion/oedema but LVEF >45%, review echo and clinical presentation with cardiologist to determine if treatment as for patients with moderate-severe LV systolic impairment is recommended

b) If clinically hypervolaemic and not on diuretics, start furosemide 40mg OD

- If already on furosemide increased dose by 40mg/d up to maximum 360mg/d

c) Address hypertension, AF, diabetes and CAD if present (see relevant recommendations)

3. If echo is of poor quality and LVEF cannot be accurately quantified, arrange contrast echo as inpatient

Specialist referral criteria

- Patients with echo report of heart failure with moderate to severe valve disease should be discussed with a cardiologist prior to initiation of new medications (see protocol for management of valve disease)
- Patients who have been established on maximally tolerated doses of beta blocker, ACE-I and spironolactone and remain symptomatic should be referred to cardiology for further specialist input (this should happen via the heart failure service)

References

1) Chronic heart failure in adults: diagnosis and management NICE guideline [NG106] Published date: 12 September 2018 https://www.nice.org.uk/guidance/ng106

2) Acute heart failure: diagnosis and management, NICE Clinical guideline [CG187] https://www.nice.org.uk/guidance/cg187

3) 2016 ESC Guidelines for the diagnosis and treatment of acute and chronic heart failure https://academic.oup.com/eurheartj/article/37/27/2129/1748921

4) British cardiology society Heart Failure with Preserved Ejection Fraction: Pathologies, Aetiology and Directions for Treatment http://www.bcs.com/documents/D38_HFpEF_Review_3.pdf
5) NICE clinical knowledge summary: Heart failure - chronic https://cks.nice.org.uk/heart-failure-chronic#!topicSummary

SCATECOPD management summary: lipids and coronary artery disease

Rationale and definitions

- Statins are likely to be required in a high percentage of patients in the SCATECOPD study, either because of known coronary artery disease (CAD) or because of high risk of future CAD-related events
- Known CAD is defined as previous myocardial infarction (MI) or coronary revascularisation
- CT calcium score ranges between 0 and 1000+
  - Scores ≥ 100 are regarded as moderate to severe and have been demonstrated to correlate with significant coronary artery stenosis and a risk benefit-ratio favouring primary prevention with aspirin

QRISK3

- Gives the likelihood of a patient without established cardiovascular disease developing it within the next 10 years
- ***Calculate for all patients*** at https://www.qrisk.org/three/index.php using admission HDL/total cholesterol level
  - If total cholesterol > 7.5 mmol/l or non-HDL cholesterol > 5.9 mmol/l QRISK3 should not be used; repeat fasting lipid profile and assess for familial hypercholesterolaemia using FATS7 strategy (see reference)

Treatment with statins

Switch any existing statin prescriptions to those recommended below. Before starting, check ALT is not > 3 x upper limit of normal and there is no unexplained muscle pain (see FATS7)

- ***Patients with known CAD should be taking atorvastatin 80mg OD***
- ***Patients without the above, but who have QRISK3 ≥ 10% should be taking atorvastatin 20mg OD***

- Additionally, patients over 40 with type 1 diabetes, or with CKD at any age (defined as albuminuria or eGFR <60ml/min/1.73m^2), should be offered atorvastatin 20mg OD regardless of QRISK3 score
- ***Patients with QRISK3 < 10% but with CT calcium score ≥ 1 should be taking atorvastatin 20mg OD***

Treatment with aspirin

- ***All patients with known CAD should be taking aspirin 75mg OD***
- ***All patients with CT calcium score ≥ 100 should be taking aspirin 75mg OD***
  - If intolerant of aspirin prescribe clopidogrel 75mg OD
- Co-prescribe lansoprazole 15mg OD if age ≥ 75 and/or history of GI bleed/peptic ulcer disease/severe gastro-oesophageal reflux disease

Follow up and referral

- Patients prescribed statins should have lipid profile repeated at their GP practice at 3-4 months, as well as LFTs
  - If non-HDL cholesterol has not fallen by > 40%, double statin dose up to maximum of 80mg atorvastatin
  - If ALT has risen to > 3x upper limit of normal statin should be stopped
- Patients should be advised to report any new muscle pains to their GP for consideration of the need for treatment cessation, dose reduction and/or measurement of creatine kinase levels
- If non-fasting triglycerides > 4.5 mmol/l a fasting sample should be taken and secondary causes excluded (e.g. uncontrolled diabetes mellitus, hypothyroidism). If fasting level > 10 mmol/l consider lipid clinic referral (see FATS7 for further advice)

References

- NICE clinical guideline [CG95]: Recent onset chest pain of suspected cardiac origin: assessment and diagnosis https://www.nice.org.uk/guidance/cg95
- NICE clinical guideline [CG181]: Cardiovascular disease: risk assessment and reduction, including lipid modification https://www.nice.org.uk/guidance/cg181
- Coronary calcium score and cardiovascular risk, Journal of the American College of Cardiology https://www.onlinejacc.org/content/72/4/434
- FATS7 cholesterol lowering strategy http://www.northoftyneapc.nhs.uk/wp-content/uploads/sites/6/2012/03/FATS7-final-updated-appendices-November-2016.pdf

SCATECOPD management summary: atrial fibrillation

Definition

Atrial fibrillation (AF) shown on ECG while in hospital or total AF burden > 5 mins on 24h ambulatory ECG monitor

Treatment goals

1. Assessment of stroke/bleeding risk and introduction of anticoagulation if appropriate
2. Control of heart rate to ≤ 110 bpm at rest
    - Use mean of multiple heart rate measurements if inpatient, otherwise single clinic measurement

Pharmacological therapy

- Send TFTs and if hyperthyroid address this first by obtaining urgent endocrinology advice on need for beta blockade e.g. with atenolol

Anticoagulation:

- Calculate CHA_2DS_2-VASc and HAS-BLED scores and review echo report for evidence of mitral stenosis
- Start anticoagulation if CHA_2DS_2-VASc score ≥ 1 in men or ≥ 2 in women, unless bleeding risk considered excessive, e.g. recent bleeding event, HASBLED score ≥ 3 with risk felt to outweigh benefit
    - If moderate-severe mitral stenosis start warfarin therapy according to local guideline
    - Otherwise start apixaban 5mg BD
        - Reduce to 2.5mg BD if creatine clearance 15-29 ml/min
        - Also reduce to 2.5mg BD if creatine > 133µmol/L is associated with
          weight < 61kg or age > 80 years
    - If eGFR < 15, do not use apixaban; offer warfarin
- Stop any antiplatelet drugs (aspirin or clopidogrel) being given for primary prevention of cardiovascular disease, or for secondary prevention after a vascular event (such as MI, stroke) more than 12 months ago
    - If on antiplatelets for a vascular event within 12 months, consult with the specialist who started the antiplatelets before stopping

Quick reference CHA_2DS_2-VASc and HAS-BLED scores (see references for full details)

CHA_2DS_2-VASc risk	Score
**C**HF or LVEF ≤ 40%	1
**H**ypertension	1
**A**ge ≥ 75	2
**D**iabetes	1
**S**troke/TIA/VTE	2
**V**ascular disease	1
**A**ge 65-74	1
**S**ex =Female	1

HASBLED risk	Score
**H**ypertension	1
**A**bnormal liver/renal function	1 or 2
**S**troke	1
**B**leeding history	1
**L**abile INR	1
**E**lderly (Age > 65)	1
**D**rugs causing [illegible]	1

Rate control:

- Start bisoprolol 2.5mg OD if HR > 110 bpm at rest
- If already on bisoprolol, increase dose by 2.5mg up to maximum 10mg OD
    - Do not increase bisoprolol if systolic BP < 90 mmHg
- If bisoprolol dose maximised, start digoxin (having corrected any hypokalaemia)
    - If in hospital, load with 0.75 to 1.5mg orally in divided doses over 24h, then give maintenance dose 125 micrograms OD (62.5 micrograms OD if eGFR < 15)
    - Out of hospital, slow loading with maintenance dose is acceptable

Follow up/specialist referral

- Refer patients started on warfarin to their local anticoagulation clinic for INR monitoring
- Review at GP should take place in 2 weeks following a change in rate control medication, for assessment of pulse rate and side effects and further dose titration if necessary

- patients on digoxin may have dose increased if rate control poor, alternatively if toxicity suspected (nausea, fatigue, blurred vision) a trough level should be checked and dose reduced if level > 1ng/ml

- Cardiology referral should be made for patients if heart rate >110 bpm on maximal digoxin and bisoprolol dose

References

- 2020 ESC Guidelines for the diagnosis and management of atrial fibrillation https://www.escardio.org/Guidelines/Clinical-Practice-Guidelines/Atrial-Fibrillation-Management
- NICE Clinical guideline CG180: Atrial fibrillation: management (2014) https://www.nice.org.uk/guidance/cg180
- BMJ Best Practice – digoxin overdose https://bestpractice.bmj.com/topics/en-us/338
- CHA_2DS_2-VASc score https://www.mdcalc.com/cha2ds2-vasc-score-atrial-fibrillation-stroke-risk
- HAS-BLED score https://www.mdcalc.com/has-bled-score-major-bleeding-risk

SCATECOPD management summary: hypertension

Diagnosis and treatment target

Blood pressure (BP) ≥ 140/90 mmHg on two occasions in final 24 hours of admission

- Only one of systolic or diastolic BP needs to exceed the threshold

- Final 24 hours of admission chosen due to lack of evidence for diagnosing hypertension during acute illness

Treatment target is below 140/90 mmHg in patients under 80 and below 150/90 in patients over 80

Management of BP during inpatient admission - ***Consider drug interactions and contraindications and use lowest risk agent***

Do not start treatment in hospital if BP less than 180/120 mmHg

- Instead refer patients with BP ≥ 140/90 mmHg on two occasions in final 24 hours of admission for ambulatory blood pressure monitoring (ABPM)

Start treatment in hospital if BP ≥ 180/120 mmHg on two occasions

- These patients should also have urine tested for dipstick haematuria and albumin:creatinine ratio.

- If they have symptoms/signs attributable to hypertensive end-organ damage, such as headache, they will need specialist inpatient management of their hypertension, usually by an endocrinologist

- If <55, or co-morbid type II diabetes or heart failure at any age, start ramipril 1.25mg OD
- If >55, or of black African or African-Caribbean family origin, start amlodipine 5mg OD

On discharge, ask GP for review within 2 weeks for BP measurement and dose titration

Uptitrate medications in patients already on antihypertensives with BP ≥ 140/90 mmHg on two occasions

- If on ACE-i, double dose, up to maximum advised in BNF
- If already on maximal ACE-i dose, double CCB dose up to maximum advised in BNF
- If already on maximal ACE-i and CCB doses, add indapamide 2.5mg OD

On discharge, ask GP for review in 2-8 weeks for BP measurement and dose titration

Acting on ABPM

≥ 14 measurements taken during usual waking hours should be used

Hypertension is confirmed if daytime average is ≥ 135/85 mmHg

- If not on any antihypertensives, start ramipril or amlodipine as described for inpatients above
- If already on antihypertensives, uptitrate medications as described for inpatients above

Ask GP for review in 2-8 weeks for BP measurement dose titration

Monitoring/Follow up

Review should take place at GP between 2-8 weeks after any change and every 3-6 months otherwise.

- Review should involve BP measurement and enquiry about adverse effects of drugs started/adjusted
- U&E should be measured within 2 weeks of any change of dose of ACE-i/diuretic
- ACE-i will need to be stopped if eGFR reduces by ≥ 25% or creatinine increases by ≥ 30%

References

- 8th Joint National Committee Guidelines for Management of High Blood Pressure in Adults https://sites.jamanetwork.com/jnc8/
- NICE guideline [NG136] Hypertension in adults: diagnosis and management https://www.nice.org.uk/guidance/ng136/chapter/Recommendations#diagnosing-hypertension
- 2018 ESC/ESH guidelines for the management of arterial hypertension https://academic.oup.com/eurheartj/article/39/33/3021/5079119#186437943
- NICE clinical guideline [CG182] Chronic kidney disease in adults: assessment and management https://www.nice.org.uk/guidance/cg182/chapter/1-Recommendations

SCATECOPD management summary: diabetes

Definitions

- ***Type 2 diabetes (T2DM)***: HbA_{1C} ≥ 48 mmol/mol detected at screening and confirmed on repeat
- ***Undertreated T2DM***: patients being treated for T2DM with confirmed HbA_{1C} ≥ 58 mmol/mol

Goals of inpatient management

- Identification and confirmation of raised HbA_{1C} levels and initiation/escalation of treatment for T2DM with involvement of inpatient diabetes services
- Shared decision making and clinical judgement regarding target HbA_{1C} levels (likely to be higher in frail patients)

There is a possibility that patients could have type 1 diabetes identified during admission. They will typically have one or more of the following: age < 50, BMI < 25, weight loss, ketosis. They should be urgently referred to diabetes specialists during inpatient stay

Acting on HbA_{1C} results in hospital

- Patients without a diagnosis of T2DM with HbA_{1C} ≥ 48 mmol/mol should have HbA_{1C} repeated in hospital
  - If T2DM confirmed: discuss lifestyle measures and initiation of metformin treatment with patient, involving inpatient diabetes service
- Patients with known T2DM with HbA_{1C} ≥ 58 mmol/mol should have HbA_{1C} repeated in hospital, provided treatment escalation has not already taken place within the last 3 months
  - If HbA_{1C} ≥ 58 mmol/mol is confirmed, escalation of therapy should be offered and referral made to inpatient diabetes service for advice and recommendations for follow up

Patients with heart disease and diabetes

- Patients with established atherosclerotic heart disease (history of MI, PCI + stent, CABG, angina) or heart failure with LV ejection fraction < 45% are likely to benefit from an SGLT2 inhibitor
  - NICE and local formulary guidance currently restricts prescribing to patients intolerant of other agents
  - Patients with the above heart conditions should nevertheless be referred to diabetes service as inpatients for consideration of switch to SGLT2 inhibitor or to plan for outpatient review to discuss this

GP Follow up

- Accurate communication in discharge letter to GP will be essential to ensure abnormal HbA_{1C} results are repeated and acted up; patients +/- relatives will also need to be fully appraised of the plan
- Patients who do have inpatient changes to medications should see their GP at 3 months for review of HbA_{1C} level, discussion of ongoing target HbA_{1C} and consideration of further treatment intensification
- Patients with HbA_{1C} 42-47 mmol/l should have HbA_{1C} repeated yearly due to high risk of progression to T2DM

References

- NICE public health guideline [PH38] Type 2 diabetes: prevention in people at high risk https://www.nice.org.uk/guidance/ph38

- The impact of corticosteroid treatment on haemoglobin A1C levels among patients with type-2 diabetes with chronic obstructive pulmonary disease exacerbation https://www.resmedjournal.com/article/S0954-6111(14)00288-1/fulltext
- NICE pathway: Type 2 diabetes in adults https://pathways.nice.org.uk/pathways/type-2-diabetes-in-adults
- NICE technology appraisal guideline [TA390] Canagliflozin, dapagliflozin and empagliflozin as monotherapies for treating type 2 diabetes https://www.nice.org.uk/guidance/ta390

Section S3: CONSORT checklist

CONSORT 2010 checklist of information to include when reporting a randomised trial*

Section/Topic	Item No	Checklist item	Reported on page No
**Title and abstract**			
	1a	Identification as a randomised trial in the title	1
	1b	Structured summary of trial design, methods, results, and conclusions (for specific guidance see CONSORT for abstracts)	2
**Introduction**			
Background and objectives	2a	Scientific background and explanation of rationale	3-5
	2b	Specific objectives or hypotheses	3-5, 7
**Methods**			
Trial design	3a	Description of trial design (such as parallel, factorial) including allocation ratio	5-6
	3b	Important changes to methods after trial commencement (such as eligibility criteria), with reasons	-
Participants	4a	Eligibility criteria for participants	6
	4b	Settings and locations where the data were collected	5
Interventions	5	The interventions for each group with sufficient details to allow replication, including how and when they were actually administered	6-7, supp. appendix
Outcomes	6a	Completely defined pre-specified primary and secondary outcome measures, including how and when they were assessed	7, supp. Appendix
	6b	Any changes to trial outcomes after the trial commenced, with reasons	-
Sample size	7a	How sample size was determined	8
	7b	When applicable, explanation of any interim analyses and stopping guidelines	-

Randomisation:			
Sequence generation	8a	Method used to generate the random allocation sequence	6
	8b	Type of randomisation; details of any restriction (such as blocking and block size)	6
Allocation concealment mechanism	9	Mechanism used to implement the random allocation sequence (such as sequentially numbered containers), describing any steps taken to conceal the sequence until interventions were assigned	-
Implementation	10	Who generated the random allocation sequence, who enrolled participants, and who assigned participants to interventions	6
Blinding	11a	If done, who was blinded after assignment to interventions (for example, participants, care providers, those assessing outcomes) and how	6
	11b	If relevant, description of the similarity of interventions	-
Statistical methods	12a	Statistical methods used to compare groups for primary and secondary outcomes	8
	12b	Methods for additional analyses, such as subgroup analyses and adjusted analyses	-
**Results**			
Participant flow (a diagram is strongly recommended)	13a	For each group, the numbers of participants who were randomly assigned, received intended treatment, and were analysed for the primary outcome	Figure 1
	13b	For each group, losses and exclusions after randomisation, together with reasons	11, Figure 1
Recruitment	14a	Dates defining the periods of recruitment and follow-up	9
	14b	Why the trial ended or was stopped	-
Baseline data	15	A table showing baseline demographic and clinical characteristics for each group	Table 1
Numbers analysed	16	For each group, number of participants (denominator) included in each analysis and whether the analysis was by original assigned groups	Figure 1; 11-13

Outcomes and estimation	17a	For each primary and secondary outcome, results for each group, and the estimated effect size and its precision (such as 95% confidence interval)	11-13, figures 2-5, Table 2
	17b	For binary outcomes, presentation of both absolute and relative effect sizes is recommended	-
Ancillary analyses	18	Results of any other analyses performed, including subgroup analyses and adjusted analyses, distinguishing pre-specified from exploratory	-
Harms	19	All important harms or unintended effects in each group (for specific guidance see CONSORT for harms)	-
**Discussion**			
Limitations	20	Trial limitations, addressing sources of potential bias, imprecision, and, if relevant, multiplicity of analyses	15
Generalisability	21	Generalisability (external validity, applicability) of the trial findings	15
Interpretation	22	Interpretation consistent with results, balancing benefits and harms, and considering other relevant evidence	13-16
**Other information**			
Registration	23	Registration number and name of trial registry	16
Protocol	24	Where the full trial protocol can be accessed, if available	16
Funding	25	Sources of funding and other support (such as supply of drugs), role of funders	16

*We strongly recommend reading this statement in conjunction with the CONSORT 2010 Explanation and Elaboration for important clarifications on all the items. If relevant, we also recommend reading CONSORT extensions for cluster randomised trials, non-inferiority and equivalence trials, non-pharmacological treatments, herbal interventions, and pragmatic trials. Additional extensions are forthcoming: for those and for up to date references relevant to this checklist, see www.consort-statement.org.
